# Telomere-to-telomere African wild rice (Oryza longistaminata) reference genome reveals segmental and structural variation

**DOI:** 10.1093/gigascience/giaf074

**Published:** 2025-08-19

**Authors:** Xuanmin Guang, Jingnan Yang, Shilai Zhang, Fei Guo, Linzhou Li, Xiaoping Lian, Tao Zeng, Chongyang Cai, Fushu Liu, Zhihao Li, Yangzi Hu, Dongming Fang, Weiming He, Sunil Kumar Sahu, Wangsheng Li, Haorong Lu, Yuxiang Li, Huan Liu, Xun Xu, Ying Gu, Fengyi Hu, Yuliang Dong, Tong Wei

**Affiliations:** State Key Laboratory of Genome and Multi-omics Technologies, BGI Research, Shenzhen 518083, China; BGI Research, Shenzhen 518083, China; BGI Research, Shenzhen 518083, China; BGI Hangzhou, CycloneSEQ Technology Co., Ltd, Hangzhou 310030, China; State Key Laboratory for Vegetation Structure, Function and Construction (VegLab), Yunnan Technology Innovation Center of Perennial Rice, New Cornerstone Science Laboratory, School of Agriculture, Yunnan University, Kunming 650091, China; BGI Research, Shenzhen 518083, China; BGI Hangzhou, CycloneSEQ Technology Co., Ltd, Hangzhou 310030, China; State Key Laboratory of Genome and Multi-omics Technologies, BGI Research, Shenzhen 518083, China; BGI Research, Wuhan 430074, China; State Key Laboratory for Vegetation Structure, Function and Construction (VegLab), Yunnan Technology Innovation Center of Perennial Rice, New Cornerstone Science Laboratory, School of Agriculture, Yunnan University, Kunming 650091, China; BGI Research, Shenzhen 518083, China; BGI Hangzhou, CycloneSEQ Technology Co., Ltd, Hangzhou 310030, China; BGI Research, Shenzhen 518083, China; BGI Hangzhou, CycloneSEQ Technology Co., Ltd, Hangzhou 310030, China; BGI Research, Shenzhen 518083, China; BGI Research, Shenzhen 518083, China; BGI Research, Shenzhen 518083, China; State Key Laboratory of Genome and Multi-omics Technologies, BGI Research, Shenzhen 518083, China; BGI Research, Shenzhen 518083, China; BGI Research, Shenzhen 518083, China; State Key Laboratory of Genome and Multi-omics Technologies, BGI Research, Shenzhen 518083, China; BGI Research, Wuhan 430074, China; BGI Research, Shenzhen 518083, China; BGI Research, Shenzhen 518083, China; BGI Research, Shenzhen 518083, China; State Key Laboratory of Genome and Multi-omics Technologies, BGI Research, Shenzhen 518083, China; State Key Laboratory of Genome and Multi-omics Technologies, BGI Research, Shenzhen 518083, China; BGI Research, Shenzhen 518083, China; BGI Research, Shenzhen 518083, China; State Key Laboratory for Vegetation Structure, Function and Construction (VegLab), Yunnan Technology Innovation Center of Perennial Rice, New Cornerstone Science Laboratory, School of Agriculture, Yunnan University, Kunming 650091, China; BGI Research, Shenzhen 518083, China; BGI Hangzhou, CycloneSEQ Technology Co., Ltd, Hangzhou 310030, China; State Key Laboratory of Genome and Multi-omics Technologies, BGI Research, Shenzhen 518083, China; BGI Research, Wuhan 430074, China

**Keywords:** Oryza longistaminata, Telomere-to-telomere (T2T) genome assembly, Comparative analysis, Valuable resource

## Abstract

Rice (*Oryza sativa*) is one of the most important staple food crops worldwide, and its wild relatives serve as an important gene pool in its breeding. Compared with cultivated rice species, African wild rice (*Oryza longistaminata*) has several advantageous traits, such as resistance to increased biomass production, clonal propagation via rhizomes, and biotic stresses. However, previous *O. longistaminata* genome assemblies have been hampered by gaps and incompleteness, restricting detailed investigations into their genomes. To streamline breeding endeavors and facilitate functional genomics studies, we generated a 331-Mb telomere-to-telomere (T2T) genome assembly for this species using a hybrid approach combining PacBio HiFi, Hi-C, and CycloneSEQ ultra-long reads, covering all telomeres and centromeres across the 12 chromosomes. This newly assembled genome has markedly improved over previous versions. Comparative analysis revealed a high degree of synteny with previously published genomes. A large number of structural variations were identified between *O. longistaminata, O. glaberrima*, and *O. sativa*. A total of 2,466 segmentally duplicated genes were enriched in cellular amino acid metabolic processes. We detected slight expansion of some subfamilies of resistance genes and transcription factors. This newly assembled T2T genome of *O. longistaminata* provides a valuable resource for the exploration and exploitation of beneficial alleles present in wild relative species of cultivated rice.

## Introduction

Rice stands as one of the world’s most essential crops, serving as a staple food source for more than half of the global population [1]. Rice breeding, particularly involving wild relatives, serves as an important gene pool and is therefore critical for global food security, for which germplasms are essential. *Oryza longistaminata* (NCBI:txid4528; 2x=2n=12), an AA genome type, thrives predominantly in the tropical regions of western Africa, often in proximity to freshwater sources and swampy areas [2]. Although it is rarely used for human consumption, this species possesses a variety of beneficial traits. Notably, it is resistant to bacterial blight, which is linked to the *Xa21* locus [3]. Furthermore, *O. longistaminata* exhibits perennial growth and an exceptional capacity for biomass production, and many efforts have been made to transfer these beneficial alleles into commercial rice varieties. In addition to contributing to breeding endeavors, *O. longistaminata* serves as a vital subject of study for investigating the genetic foundations and developmental aspects of rhizomes [4].

The assembly of a complete plant genome provides a solid basis for functional genomics investigations and facilitates the identification of candidate genes via traditional mapping techniques. Despite the publication of several genome assembly versions, limitations stemming from sequencing technology and the intricate organization of the genome have left certain complex regions underrepresented [5, 6]. To achieve a more comprehensive representation of this fundamental reference genome, we employed a hybrid assembly strategy using Pacbio HiFi and CycloneSEQ ultra-long reads (a new single-molecule sequencer from MGI) [7] to generate backbone contigs. This marks one of the first applications of CycloneSEQ in plant telomere-to-telomere (T2T) genomes, demonstrating its utility for complex genomic regions [7, 8]. These contigs were subsequently scaffolded into a chromosome-level assembly with the assistance of Hi-C datasets. In addition, gap filling was executed to resolve any remaining gaps. To this end, we generated a T2T assembly for *O. longistaminata*, which could serve as a valuable genomic resource for future rice research and breeding.

## Results and Discussion

### Genome assembly

We initially sequenced the genome of *O. longistaminata*, generating 27.3 Gb (∼78× coverage) of PacBio HiFi reads, 32 Gb (∼100× coverage) of Hi-C paired reads, 25.6 Gb (∼71.4× coverage) of ultra-long CycloneSEQ reads, and 21.0 Gb (∼60× coverage) of MGI-seq paired-end reads (Supplementary Table S1). Using the *k-*mer method [9], we estimated the genome size of this plant to be 357 Mb, and its heterozygosity was 1.27% (Supplementary Fig. S1), which is similar to the size reported in previous studies [6]. Using the combined data, we first assembled a genome with a size of 343 Mb and a contig N50 of 26.02 Mb. Based on the Hi-C data, we anchored 13 contigs into 12 pseudochromosomes (Table 1, Supplementary Fig. S2). After that, TGS-gapcloser was employed to close the remaining gaps [10]. Finally using the 7-base telomeric repeat (CCCTAAA at the 5′ end or TTTAGGG at the 3′ end) as a sequence query, we identified all the 24 telomeres for the genome (Supplementary Fig. S3) [11, 12]. We then used quarTeTes to identify the centromeric regions ranging from 0.3 to 1.8 Mb on each chromosome and assessed the regions using Hi-C data [13]. Different methods were used to evaluate the accuracy and completeness of the assembly. First, paired-end library reads were mapped to the genome, and more than 97.27% of them were aligned. Second, the BUSCO analysis indicated that the completeness of the genome reached 98.6% [14] (Supplementary Table S2). Third, the LTR assembly index (LAI) value for the genome was 20.71, meeting the gold standard for genome assemblies [15]. Fourth, the calculated assembly consensus quality value (QV) using Merqury was 52.08, which indicates that the base call accuracy of the genome was greater than 99.999% [16]. As there is a previously published *O. longistaminata* genome assembly [5], we conducted a comparative gene synteny analysis at all coding sequence (CDS) levels. We subsequently identified a total of 28,627 syntenic CDS pairs through genome-wide alignment. Consistent with expectations, these 2 genomes displayed rather high synteny, as evidenced by the pronounced central diagonal in the alignment (Supplementary Fig. S4). This result demonstrated the high concordance between our assembled T2T genome and the previous *O. longistaminata* assembly.

**Table 1:** Summary statistics of the *O. longistaminata* genome assembly

Genome feature	Value
Total size of assembled contigs (bp)	343,752,306
GC content	43.02%
Contig N50	26,021,309
Number of contigs	197
Total size of assembled genomes (bp)	331,045,917
Scaffold N50	26,021,309
Complete BUSCOs	98.6%
LAI value	20.71
Repeat region	40.73%
Number of gap-free chromosomes	12
Number of candidate telomeres	24
Number of candidate centromeres	12
Number of chromosomes	12
Number of protein-coding genes	33,177
Average gene length (bp)	2,439
Average exon length (bp)	261
Average intron length (bp)	384

### Genome annotation

**Figure 1:**
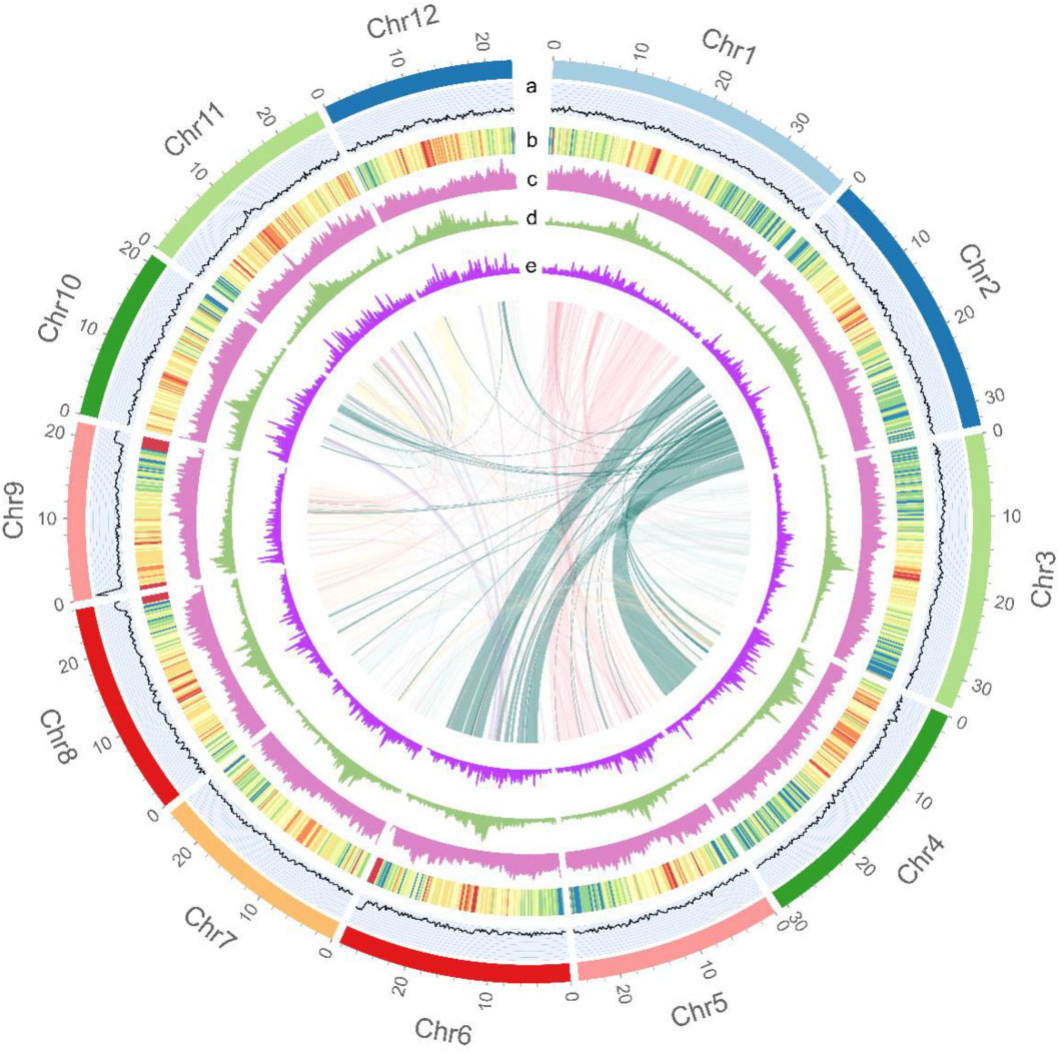
The telomere-to-telomere genome assembly of *O. longistaminata*. Genomic features of the *O. longistaminata* genome: (a) GC percentage, (b) protein-coding genes, (c) repeat sequences, (d) LTR-*Gypsy*, and (e) and LTR-*Copia*. The collinear blocks are shown in the center.

Through the utilization of *de novo* and homology-based methods, we successfully identified a total of 134 Mb of repetitive sequences in the plant genome. These repetitive sequences make up approximately 40.73% of the entire genome. Furthermore, we observed that the repeat contents were highly consistent across the entire genome, as well as among the 12 pseudochromosome sequences (Fig. 1, Supplementary Table S3). In this genome, LTRs and DNA transposons were the major types of repeats, accounting for approximately 20.9% and 18.5% of the whole genome, respectively. The overall repeat content was moderate, similar to the repeat content observed in other assemblies with *Oryza* genus genomes [17].

The centromere region of the genome poses a significant challenge for assembly because of its high degree of repetitive sequence content [18]. To date, the centromeric sequence of the *O. longistaminata* genome has not been fully characterized, and our new T2T genome allows deeper exploration of the repeats in these regions. The results revealed a phenomenon in which centromeric regions presented high densities of transposable elements and relatively low gene densities. Among the repeats of the centromeric regions, Gypsy elements were the most dominant type of LTR (Supplementary Fig. S5).

A total of 33,177 coding genes were predicted in this genome, with an average gene length of 2,439 bp and an average CDS length of 1,138 bp (Fig. 1, Table 1, Supplementary Table S4). The functional analysis revealed that 95.74% of the coding genes could be annotated through publicly available protein datasets (Supplementary Table S5), suggesting the accuracy of gene prediction.

### Genome structural variations

**Figure 2:**
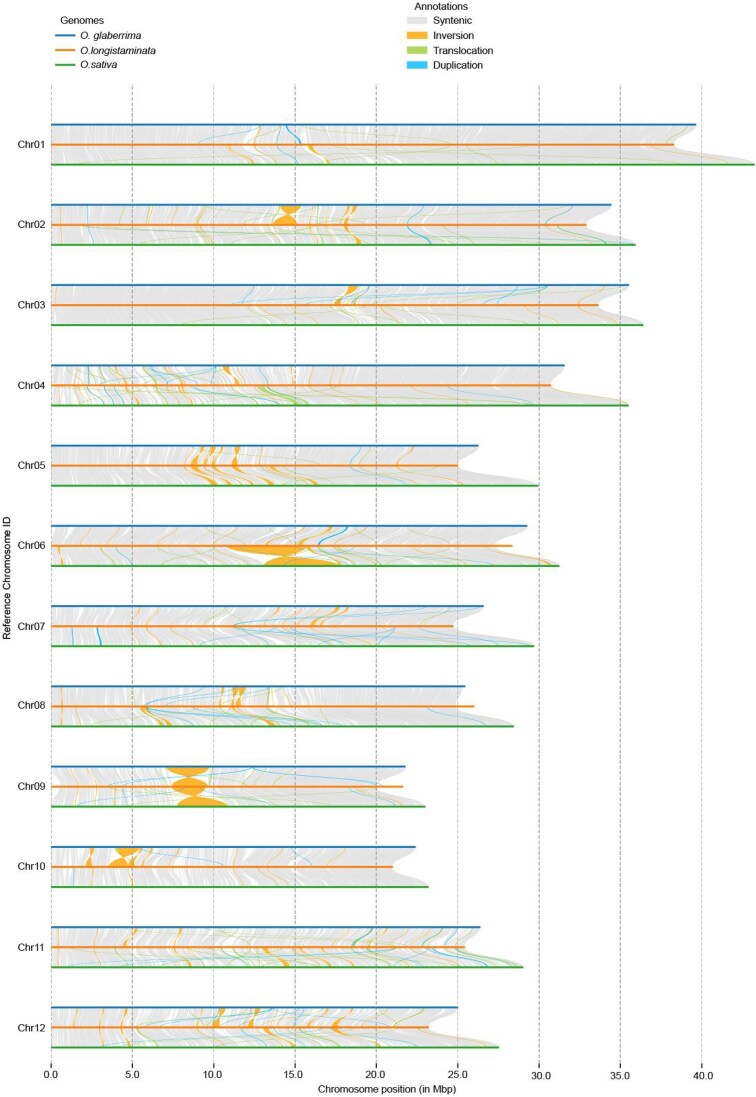
Collinearity and variation analysis of the T2T genome of *O. longistatminata, O. sativa* Japonica, and *O. glaberrima*. The orange, blue, and green lines indicate *O. longistatminata, O. glaberrima*, and *O. sativa* Japonica, respectively. Gray and blank blocks between various genomes indicate syntenic regions and not-aligned regions. Inversions, translocations, and duplications are marked by filled orange, green, and blue curves.

We further performed genome-wide detection of putative structural variations (SVs) with the *O. glaberrima* (IRGC:96,717) genome (Fig. 2). A total of 4,790,440 single-nucleotide polymorphisms (SNPs) were identified by comparing the 2 genomes. Among the SVs identified in our study, there were 198 inversions, 8,263 duplications, 8,093 inverted duplications, 2,667 translocations, and 2,663 inverted translocations (Supplementary Table S6). These large SVs span more than 87 Mb throughout the entire genome, which indicates remarkable divergence between these 2 species. Gene Ontology (GO) enrichment analysis of these SV-related genes revealed that they were associated with adenosine diphosphate (ADP) binding, transposition, transposase activity and transposition, and DNA-mediated (Supplementary Table S7). Furthermore, we conducted a comparative analysis with the *O. sativa* Japonica genome for SV identification (Fig. 2). There were 3,738,150 SNPs, 204 inversions, 11,706 duplications, 11,175 inverted duplications, 3,077 translocations, and 3,015 inverted translocations (Supplementary Table S8). GO enrichment of these SV affected genes was related to catalytic activity, purine ribonucleotide binding, adenyl ribonucleotide binding, and telomere maintenance (Supplementary Table S9).

### Analysis of segmental duplications in the genome

**Figure 3:**
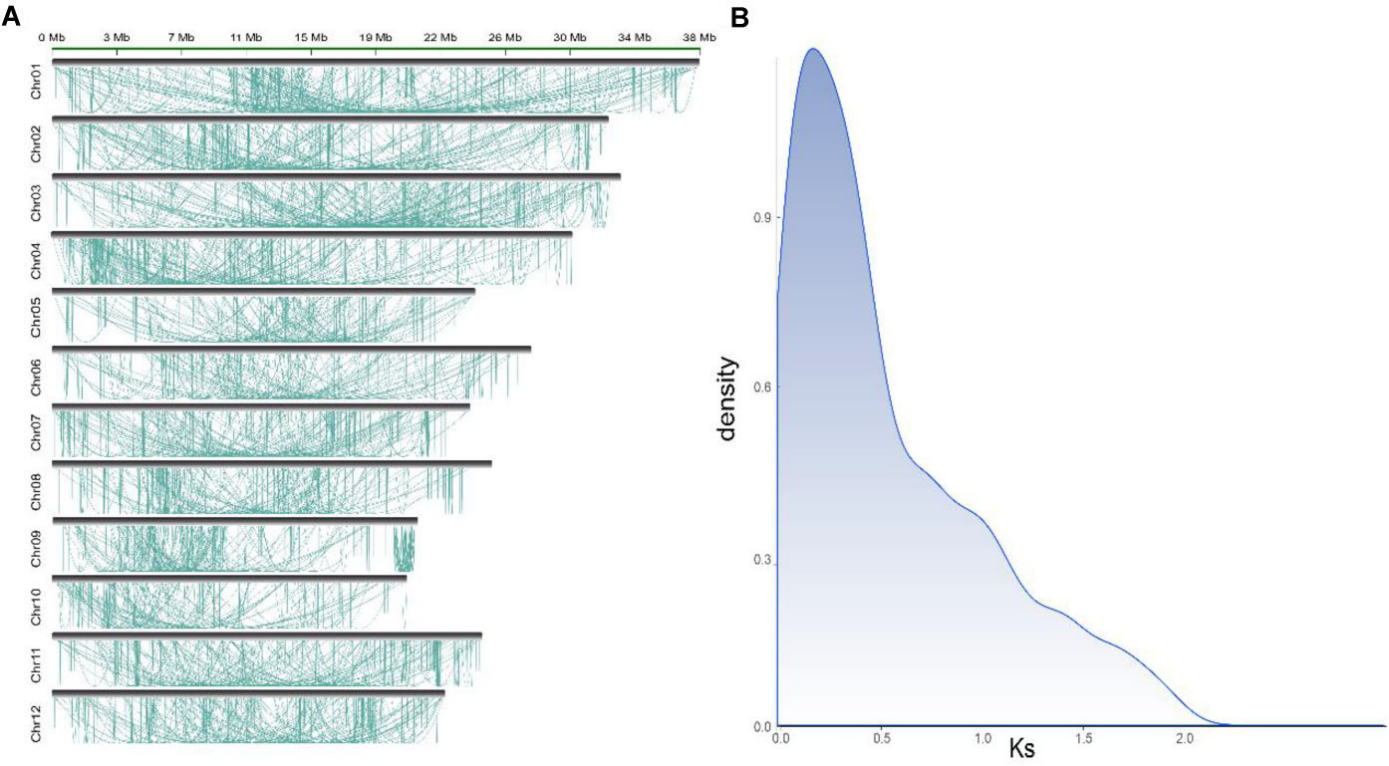
Segmental duplication analysis of the genome of *O. longistaminata*. (A) Distribution of intrachromosomal segmental duplication. (B) The density plot of the *Ks* value.

Segmental duplications (SDs) are genomic segments larger than 1 kb that repeat within the genome and exhibit at least 90% sequence identity [19]. SDs frequently contain numerous duplicated genes, making them vital centers for gene innovation. Challenges in assembly technology have caused the assembly of SD regions to collapse or be entirely overlooked. As a result, this missing or inaccurate information limits our ability to understand the structure and evolution of a genome. The T2T genome of *O. longistaminata* offers an opportunity for more accurate characterization of SDs. In this study, we employed BISER to analyze the SDs in the rice genome [20], identifying 30.2 Mb of SDs, which constitute 9.12% of the genome. We discovered that SDs are not evenly distributed throughout the genome (Fig. 3A). Instead, they are more frequently found on chromosomes 1 (chr1), 4 (chr4), 3 (chr3), and 2 (chr2) and less frequently on chromosomes 9 (chr9), 10 (chr10), and 5 (chr5). Correlation analysis revealed a strong positive relationship between chromosome length and SDs (*R* = 0.88, *P* = 0.00017) (Supplementary Table S10).

We proceeded to identify duplicated genes within the SD regions. Initially, we conducted an all-versus-all alignment via BLASTP [21] to identify potential paralogs, setting an E-value threshold of 10^–5^. In the SD regions, we identified a total of 4,179 pairs, of which 1,233 were the top matches. For each paralogous gene pair within the SD regions, we calculated their *Ks* values as proxies for estimating the generation time of the corresponding SDs. Our findings indicate that most of these SDs were produced relatively recently (*Ks* = 0.3) (Fig. 3B). GO analysis revealed that these genes were significantly enriched (*P* < 0.05) in cellular amino acid metabolic processes, carboxylic acid metabolic processes, and cofactor binding (Supplementary Table S11).

### NBS gene family and transcription factors

Nucleotide-binding site-leucine-rich repeat (NBS-LRR) proteins, the largest family of resistance proteins, are very important for plant defense against pathogens [22, 23]. We systematically investigated the NBS-LRR genes among 11 *Oryza* species (*O. barthii, O. brachyantha, O. glaberrima, O. glumipatula, O. indica, O. meridionalis, O. nivara, O. punctata, O. rufipogon, O. sativa, O. longistaminata*) (Supplementary Table S12). There were 654 NBS-LRR genes in the *O. longistaminata* genome, which were distributed in 5 different clusters (Supplementary Table S13). Compared with other wild *Oryza* species, *O. longistaminata* has more NBS-LRR domain genes, which reflects the expansion of resistance genes in this species. NBS-LRR genes are essential components of the plant immune system, providing a mechanism for pathogen recognition and the activation of defense responses. The expansion of these types of genes in *O. longistaminata* may suggest that there was an increased ability for this species to adapt to its evolution.

We also investigated the variation in transcription factors among *Oryza* species. For *O. longistaminata*, a total of 2,095 transcription factors were distributed among 86 families (Supplementary Table S14). The ERF transcription factor was the most abundant (857), followed by the bHLH family (128), NAC (120), MYB (119), and C2H2 (116). Intriguingly, we found that there were 47 FAR1 genes in *O. longistaminata*, which was much larger than those in other African rice accessions. Research has shown that *FAR1* performs various functions across numerous cellular processes, indicating that *FAR1* is crucial for plant growth and development [24].

## Conclusion

In this study, we generated a high-quality T2T assembly of wild rice *O. longistaminata* using an integrated approach that leveraged CycloneSEQ ultra-long reads to resolve complex repetitive regions, particularly centromeres and telomeres. This hybrid strategy, combining CycloneSEQ with PacBio HiFi accuracy and Hi-C scaffolding, enabled the first complete assembly of all 24 telomeres and 12 centromeres in this species. We further compared this assembly with previously published *Oryza* genomes and identified SVs between 2 African wild rice accessions and *O. sativa*. Moreover, we investigated SD genes, NBS-LRR resistance genes, and transcription factors. This new *O. longistaminata* assembly represents a significant update, laying fundamental evidential groundwork for focused investigations into genes associated with valuable phenotypic traits. It also sets the stage for future breeding endeavors, as well as further exploration into the evolutionary pathways of African rice and the *Oryza* genus.

## Methods

### Material preparation and sequencing

Fresh young leaves from mature *O. longistaminata* plants were collected from Yunnan University, Yunnan Province, China. Genomic DNA was extracted for PacBio HiFi, MGI CycloneSeq, and MGIseq (DNBSEQ-T7) (RRID:SCR_017981). Pair-end libraries 500 bp in length were constructed and sequenced on the MGIseq platform. For Cyclone sequencing, genomic DNA was extracted via the CTAB method [25], and the CycloneSEQ library was generated following the manufacturer’s guidelines. Each sample, comprising 2 μg input DNA (≥21 ng/μL), was initially diluted with nuclease-free water to a total volume of 192 μL, followed by mixing with 14 μL DNA repair buffer 1, 14 μL DNA repair buffer 2, 12 μL DNA repair enzyme 1, and 8 μL DNA repair enzyme 2. The mixtures were then incubated in a thermocycler through the following steps: 10 minutes at 20°C, 10 minutes at 65°C, and held at 4°C. After incubation, the mixes were purified using a 1.0× volume of DNA clean beads, and DNA were eluted with 240 μL nuclease-free water. Next, the purified end-repaired samples were mixed with 10 μL sequencing adaptors, 100 μL 4× ligation buffer, 40 μL DNA ligase, and 10 μL nuclease-free water before being incubated at 25°C for 30 minutes to complete the adaptor ligation. The ligated products were again purified with a volume of 1.0× DNA clean beads, and long fragment wash buffer was applied to gently resuspend the beads. After removing the supernatant, the libraries were recovered in 42 μL elution buffer and quantified on a Qubit fluorometer [26]. Each prepared library was sequenced on the CycloneSEQ (WuTong02) platform according to the protocol [27]. A total of 25.6 Gb of clean subreads longer than 79 kb were obtained and used as ultra-long reads. For the construction of the PacBio HiFi library, more than 5 µg DNA was prepared for size selection using a BluePippin (RRID:SCR_020505) instrument. Subsequently, PacBio Sequel II single-molecule real-time (SMRT) bell libraries of approximately 20 kb were constructed in accordance with the PacBio protocol. The library was loaded into SMRT cells with a DNA Sequencing Reagent Kit. These SMRT cells were then run on a PacBio Sequel II CCS system (RRID:SCR_017990), which generated 24 Gb of long-read sequencing data.

### Genome assembly

We employed hifiasm (RRID:SCR_021069, v 0.19.5-r592) for genome assembly, utilizing both HiFi reads and ultra-long CycloneSeq reads under the mixed assembly model with default settings [28]. Subsequent polishing of the assembled genome was performed using NextPolish (RRID:SCR_025232) [29] with MGISEQ reads (RRID:SCR_017981). For chromosomal anchoring of the contigs, we first utilized cleaned HiC reads. Unique mapping reads were identified using bowtie2 (RRID:SCR_016368, v 2.3.2) [30], followed by the detection of valid interacting paired reads via Juicer (RRID:SCR_017226, v 2.8.1) [31]. These valid read pairs were then used to construct pseudo-chromosome sequences with 3D-DNA [32]. The HiC interactions were shown as heatmaps through Juicebox (RRID:SCR_021172) [33]. After that, the genome had only 1 gap. Finally, gap filling was accomplished by using TGS-Gapcloser (RRID:SCR_017633, v1.2.1) [10] with CycloneSeq reads, and corrections were made by using pilon (RRID:SCR_014731, v 1.24) with MGI paired-end reads [34]. Based on the genome of *O. sativa*, we artificially reoriented some chromosomes and renamed their chromosome numbers.

To access the quality of the assemblies, we mapped the short paired-end reads to the assembly by using the BWA-MEM tool from BWA (RRID:SCR_022192) [35], and BUSCO analysis was performed with the embryophyte_odb9 database (RRID:SCR_015008) [14].

### Genome annotation

We initially generated repetitive libraries for both species through a dual approach involving homology comparison and *de novo* prediction. Repetitive sequences were identified using LTR Finder (RRID:SCR_015247) [36] and RepeatModeler (RRID:SCR_015027) [37]. Homology-based prediction was performed using TRF (RRID:SCR_022193) [38] and RepeatMasker (RRID:SCR_012954) [39] with the Repbase TE library. The annotated and classified repetitive sequences were then used to mask the genomes with RepeatMasker. Additionally, we employed LTR Retriever [40], in conjunction with LTR Finder, to calculate the LAI, which assesses assembly continuity by evaluating the assembly of repeat sequences [15].

For the RNA-seq assisted predictions, ISO-seq sequences were obtained from a mixed tissue. We employed SMRT Link v8.0, applying the parameters –min-passes 3, –min-length 50, –max-length 15,000, and –min-rq 0.99 to refine the circular consensus sequence (CCS) subreads, subsequently gathering high-quality reads. For classifying the full-length reads, Lima (RRID:SCR_025520, v2.2.0) was utilized with the following settings: –isoseq, –dump-clips, and –peak-guess [41]. The final assembly of full-length Iso-seq transcripts was achieved using isoseq3 (RRID:SCR_022749) [42], which employs the refine module (parameters: –require-polya and –min-polya-length 20) and the cluster module (parameters: –verbose and –use-qvs). After that, Transdecode (RRID:SCR_017647) [43] was used to predict the CDS, and the longest CDSs were fed into Maker for gene annotation. For homologous predictions, protein sequences from *Zea mays, O. sativa*, and *Arabidopsis thaliana* were used. The MAKER2 (RRID:SCR_005309) pipeline [44] was used for protein-coding gene annotation, and *de novo* gene models were accessed via AUGUSTUS (RRID:SCR_008417) [45] and Fgenesh (RRID:SCR_011928) [46]. To predict gene functions, we performed a BLAST search of their protein sequences against the Swiss-Prot and NR databases, using a threshold E-value of 1e-5. We subsequently employed InterProScan (RRID:SCR_005829) to annotate motifs and domains by searching for matches in those databases [47].

### Structural variation analysis

We utilized a suite of tools from MUMmer4 (RRID:SCR_018171) [48] to analyze genomic differences between *O. longistaminata* and *O. glaberrima*. The nucmer tool was employed to compare syntenic chromosomes, and the results were subsequently filtered through a delta-filter with the parameters “-c 100 -b 500 -l 50.” The alignments were then converted into tab-delimited files using the show-coords program. Finally, SyRI (RRID:SCR_023008) was applied to identify SVs [49].

### Identification of telomeres and centromeres

In most plants, telomere sequences consist of short, conserved satellite repeats arranged in tandem. We identified a typical plant telomere sequence (CCCTAAA at the 5′ end or TTTAGGG at the 3′ end) and subsequently identified telomeres across all 12 chromosomes by using tidk [12]. To detect centromeric regions, we employed the quartet (RRID:SCR_025258) tool [13]. This tool is well suited for identifying genomic areas with high and low gene densities, as well as short tandem repeats, which are characteristic features of centromeric regions.

### Detection of SDs

Briefly, our genome assembly underwent an initial soft-mask process, during which all common and tandem repeats were converted into lowercase letters. Following this, BISER was employed to detect SDs, utilizing its default parameters [20].

### *Ks* of the duplicated gene pairs

Protein sequences from duplicated gene pairs within SDs were extracted and subsequently aligned utilizing the MUSCLE (RRID:SCR_011812) alignment program [50]. These aligned protein sequences were then transformed into corresponding coding sequence alignments through PAL2NAL [51]. Next, we calculated the rate of synonymous substitutions per synonymous site (*Ks*) for each gene pair, employing the KaKs_Calculator (RRID:SCR_022068) [52]. The distribution of *Ks* values was graphically plotted and visualized using the R statistical software.

### Identification of *NBS-LRR* genes and *TF* genes

For each species analyzed, protein sequences were extracted and subsequently screened against the raw hidden Markov model (HMM) of the NB-ARC family (PF00931) utilizing HMMER (RRID:SCR_005305, v 3.1b1), applying the default parameters [53]. NBS-specific HMMs were constructed utilizing the hmmbuild program within HMMER, which were then employed to identify NBS-encoding proteins. To identify specific protein domains, PfamScan (RRID:SCR_004726) was utilized for screening these proteins against the Pfam-A database (Pfam31.0) [54]. Additionally, coiled-coil domains were detected using the ncoils tool [55], with its default parameters applied. The iTAK (Integrated Toolkit for Analysis of Kinase) software tool was used to identify transcription factors and protein kinases among the *Oryza* genus species [56].

## Additional Files

**Supplementary Fig. S1**. The *k*-mer distribution of the *O. longistaminata* genome. *k*-mer = 17.

**Supplementary Fig. S2**. Heatmap for Hi-C assembly of the *O. longistaminata* genome.

**Supplementary Fig. S3**. Overview of the genome structure.

**Supplementary Fig. S4**. Homologous dot plot between T2T genome and previous *O. longistaminata* genome.

**Supplementary Fig. S5**. The length of the centromere and the repeat content of its distribution.

**Supplementary Table S1**. Summary of the sequence data for *O. longistaminata*.

**Supplementary Table S2**. BUSCO analysis of *O. longistaminata*.

**Supplementary Table S3**. Statistics of repetitive sequences in the genome.

**Supplementary Table S4**. Statistics of gene elements items.

**Supplementary Table S5**. Statistics for gene function annotation.

**Supplementary Table S6**. Summary of structural variations between *O. longistaminata* and *O. glaberrima*.

**Supplementary Table S7**. GO analysis of SV-related genes between *O. longistaminata* and and *O. glaberrima*.

**Supplementary Table S8**. Summary of structural variations between *O. longistaminata* and *O. sativa*.

**Supplementary Table S9**. GO analysis of SV-related genes between *O. longistaminata* and *O. sativa japonica*.

**Supplementary Table S10**. Statistical distribution of SDs on chromosomes.

**Supplementary Table S11**. GO analysis of SD-related genes in *O. longistaminata*.

**Supplementary Table S12**. The accession number of the *Oryza* genus genome.

**Supplementary Table S13**. Statistics of the NBS gene family among *Oryza* genus species.

**Supplementary Table S14**. Comparison of transcription factors of the *Oryza* genus.

giaf074_Supplementary_Files

giaf074_Authors_Response_To_Reviewer_Comments_Original_Submission

giaf074_Authors_Response_To_Reviewer_Comments_Revision_1

giaf074_GIGA-D-24-00479_Original_Submission

giaf074_GIGA-D-24-00479_Revision_1

giaf074_GIGA-D-24-00479_Revision_2

giaf074_Reviewer_1_Report_Original_Submission Chengzhi Liang -- 11/29/2024

giaf074_Reviewer_2_Report_Original_Submission Francois Sabot -- 12/19/2024

giaf074_Reviewer_2_Report_Revision_1 Francois Sabot -- 4/3/2025

## Abbreviations

ADP: adenosine diphosphate; BUSCO: Benchmarking Universal Single-Copy Orthologs; CCS: circular consensus sequence; CDS: coding sequence; GO: Gene Ontology; HMM: hidden Markov model; LAI: LTR assembly index; NBS-LRR: nucleotide-binding site-leucine-rich repeat; QV: quality value; SD: segmental duplication; SMRT: single-molecule real-time; SNP: single-nucleotide polymorphism; SV: structural variation; T2T: telomere-to-telomere.

## Data Availability

The T2T genome assembly and CycloneSeq reads have been deposited to NCBI and CNSA (CNGB Nucleotide Sequence Archive) with BioProject accessions PRJNA1259753 and CNP0005176, respectively. All additional supporting data are available in the *GigaScience* repository, GigaDB [57].
