# Supplementary material for: Telomere-to-telomere African wild rice (Oryza longistaminata) reference genome reveals segmental and structural variation
Source: Gigascience. 2025 Aug 19;14:giaf074. doi: 10.1093/gigascience/giaf074 (PMC12360840; doi:10.1093/gigascience/giaf074)
Supplement: giaf074_Supplementary_Files [file giaf074_supplementary_files.zip › Supplementary Figures.docx]

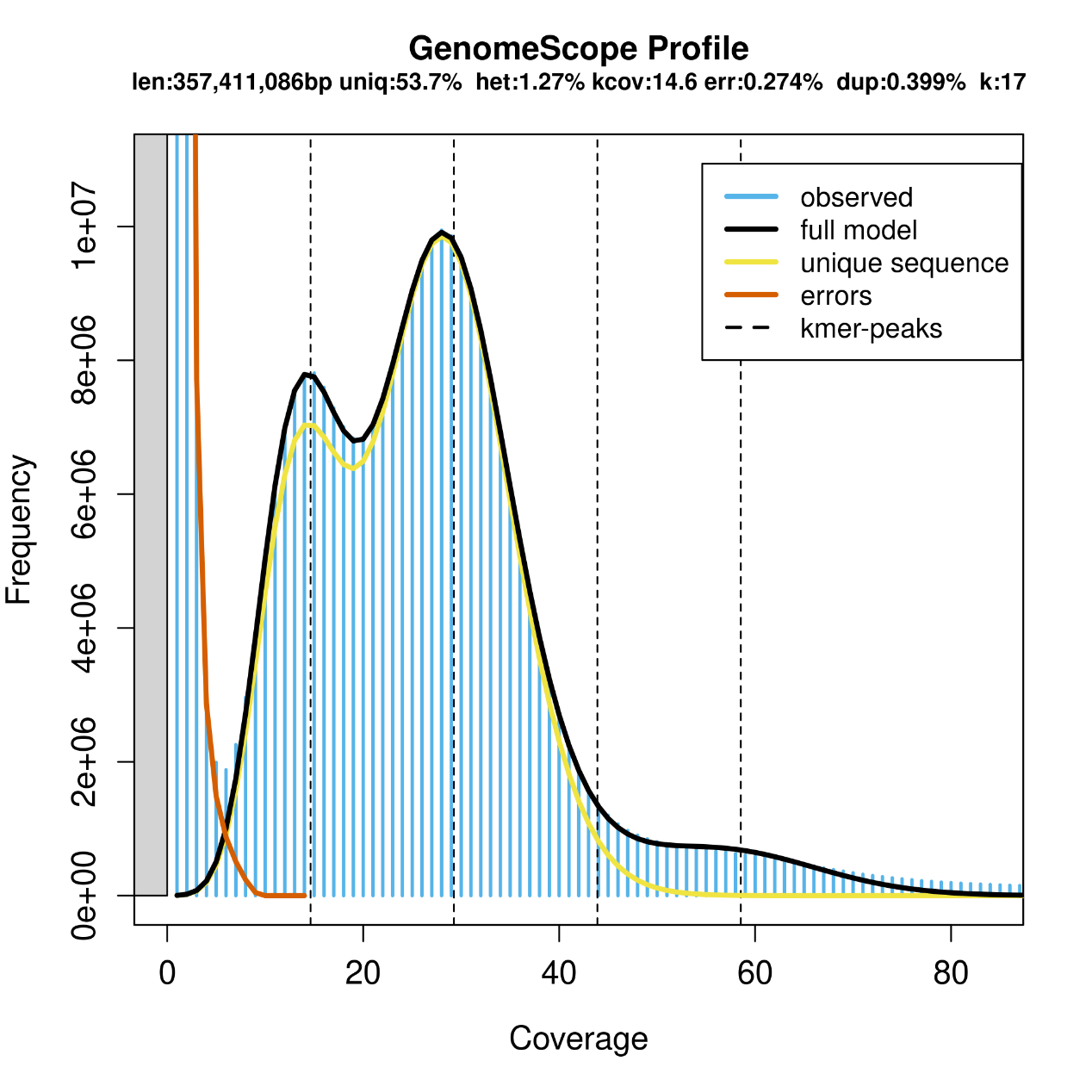


**Figure S1. *K*-mer distribution of the *O.longistaminata* genome. *K*-mer=17.**


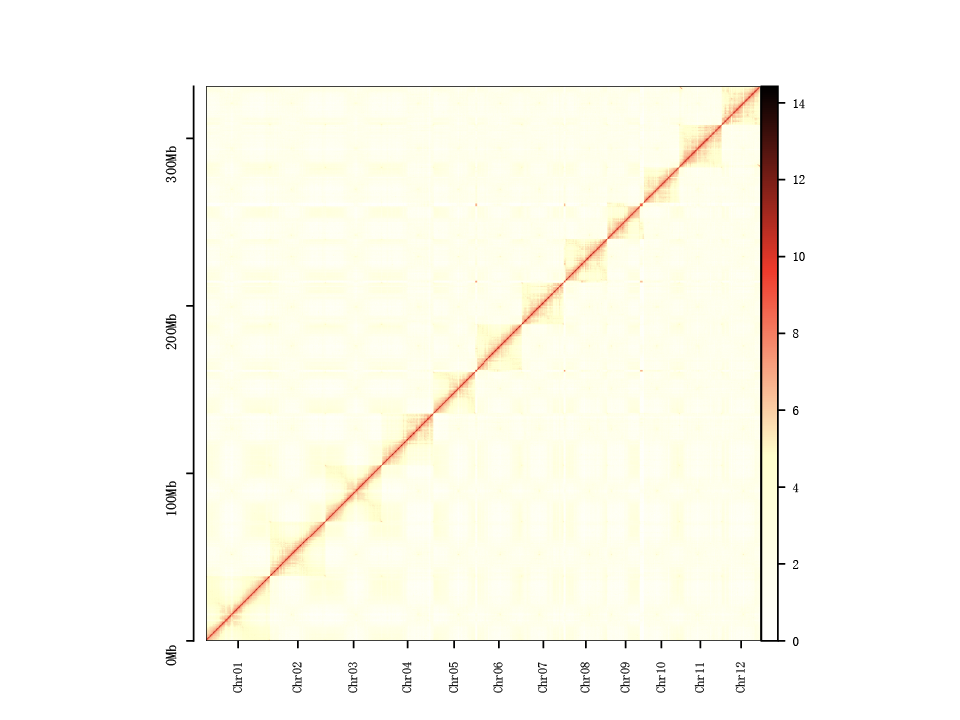


**Figure S2. Heatmap for Hi-C assembly of *O.longistaminata* genome.**


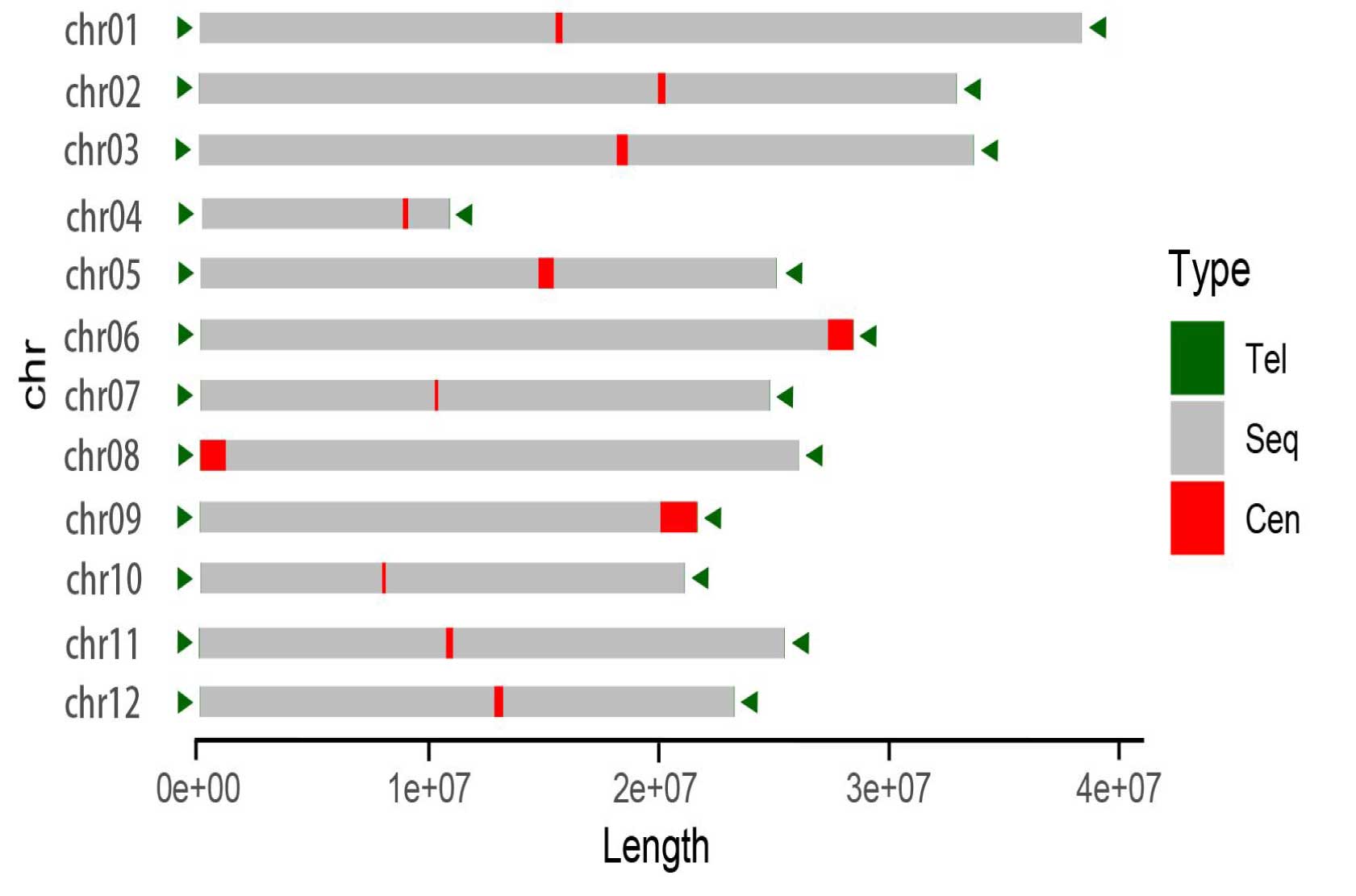


**Figure S3. The overview of the genome structure.**


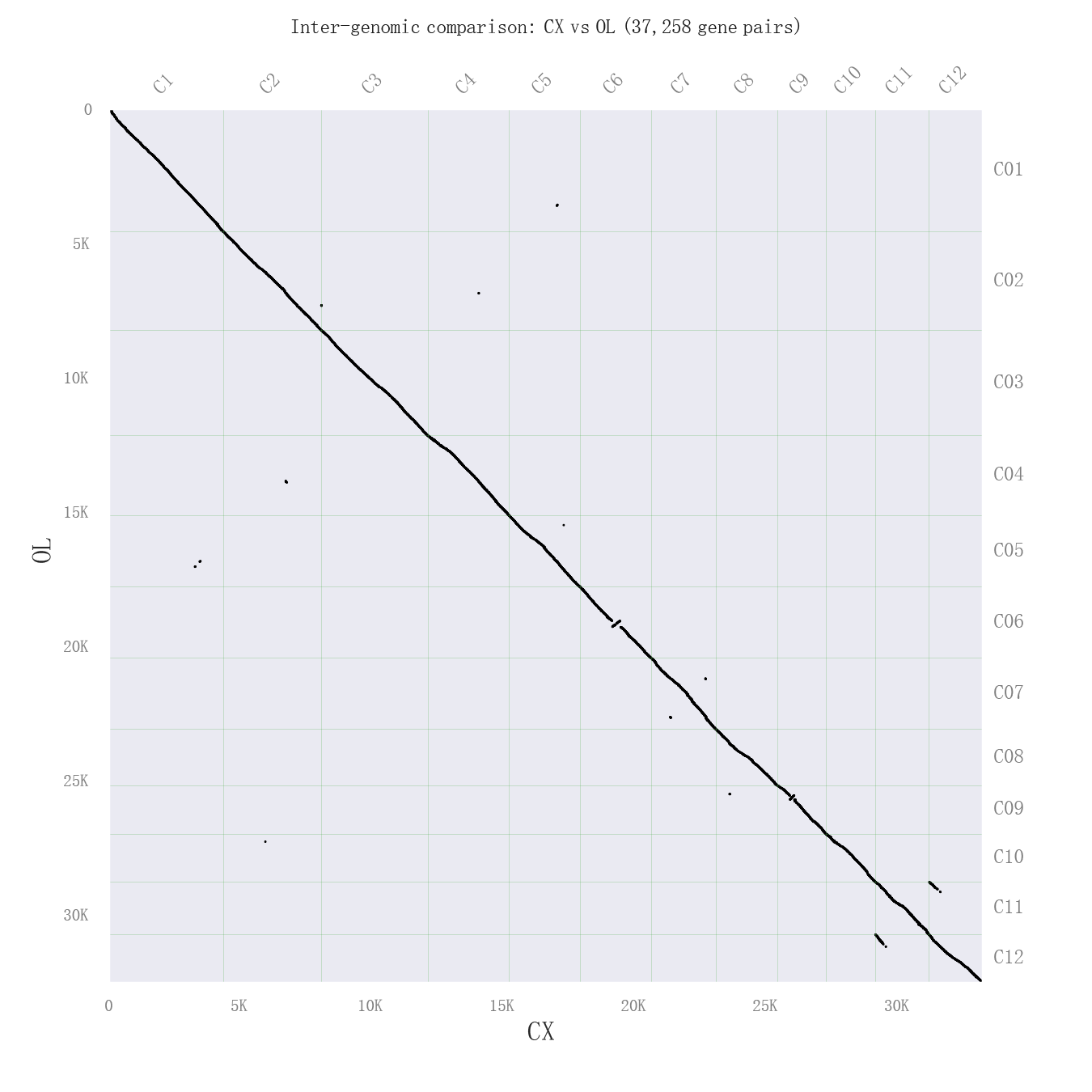


**Figure S4. Homologous dot-plot between T2T genome and previous *O.longistaminata* genome genomes.**


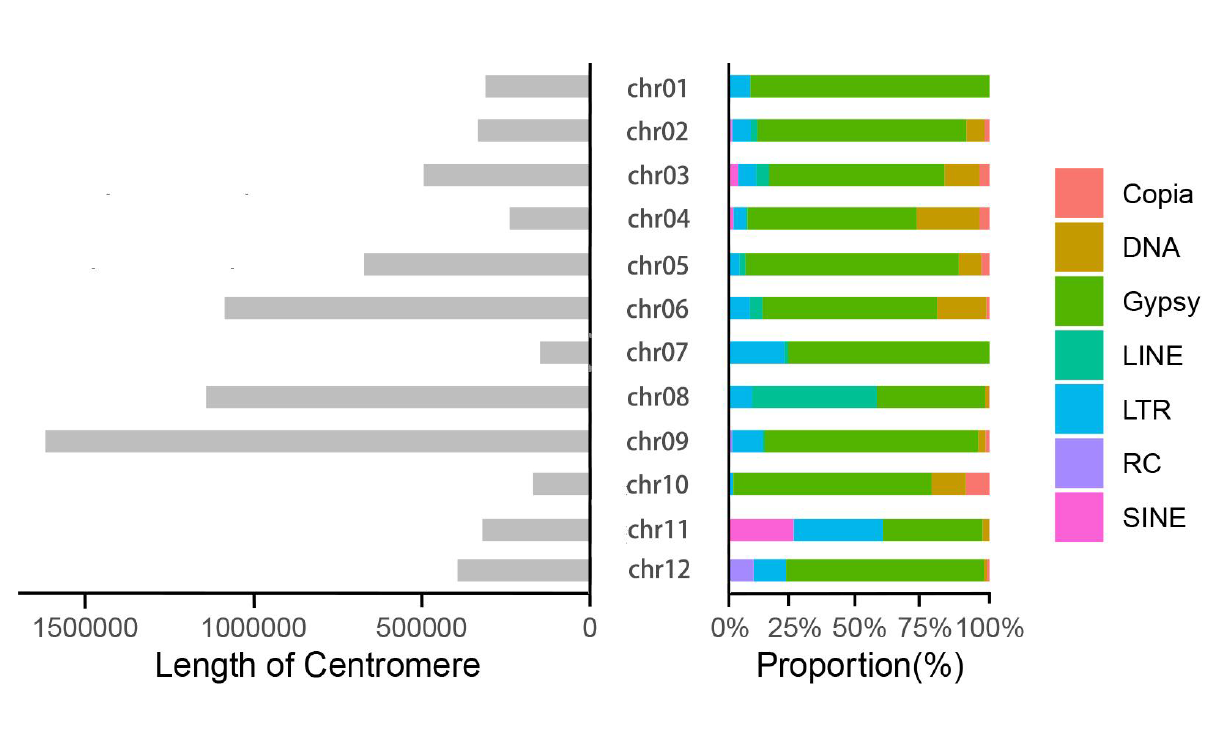


**Figure S5. The length of Centromere and the repeat content of its distribution**
