# Supplementary material for: Telomere-to-telomere African wild rice (Oryza longistaminata) reference genome reveals segmental and structural variation
Source: Gigascience. 2025 Aug 19;14:giaf074. doi: 10.1093/gigascience/giaf074 (PMC12360840; doi:10.1093/gigascience/giaf074)
Supplement: giaf074_GIGA-D-24-00479_Revision_2 [file giaf074_giga-d-24-00479_revision_2.pdf]

GigaScience

Telomere-to-telomere African wild rice (Oryza longistaminata) reference genome reveals segmental and structural variation

--Manuscript Draft--

**Manuscript Number:**	GIGA-D-24-00479R2
**Full Title:**	Telomere-to-telomere African wild rice (Oryza longistaminata) reference genome reveals segmental and structural variation
**Article Type:**	Data Note
**Funding Information:**	Science, Technology and Innovation Commission of Shenzhen Municipality (KQTD20221101093603011) — Dr Jingnan Yang<br>National Natural Science Foundation of China (32322063) — Dr Silai Zhang
**Abstract:**	Rice (Oryza sativa) is one of the most important staple food crops worldwide, and its wild relatives serve as an important gene pool in its breeding. Compared with cultivated rice species, African wild rice (Oryza longistaminata) has several advantageous traits, such as resistance to increased biomass production, clonal propagation via rhizomes, and biotic stresses. However, previous O. longistaminata genome assemblies have been hampered by gaps and incompleteness, restricting detailed investigations into their genomes. To streamline breeding endeavors and facilitate functional genomics studies, we generated a 331-Mb telomere-to-telomere (T2T) genome assembly for this species, covering all telomeres and centromeres across the 12 chromosomes. This newly assembled genome has markedly improved over previous versions. Comparative analysis revealed a high degree of synteny with previously published genomes. A large number of structural variations were identified between O. longistaminata, O. glaberrima and O.sativa. A total of 2,466 segmentally duplicated genes were enriched in cellular amino acid metabolic processes. We detected slight expansion of some subfamilies of resistance genes and transcription factors. This newly assembled T2T genome of O. longistaminata provides a valuable resource for the exploration and exploitation of beneficial alleles present in wild relative species of cultivated rice.
**Corresponding Author:**	Tong Wei, Ph.D.<br>BGI-Shenzhen: BGI Group<br>Shenzhen, --- Select One -- CHINA
**Corresponding Author Secondary Information:**	
**Corresponding Author's Institution:**	BGI-Shenzhen: BGI Group
**Corresponding Author's Secondary Institution:**	
**First Author:**	Tong Wei, Ph.D.
**First Author Secondary Information:**	
**Order of Authors:**	Tong Wei, Ph.D.<br>Xuanmin Guang, PhD<br>Jingnan Yang, PhD<br>Silai Zhang<br>Fei Guo<br>Linzhou Li, PhD<br>Xiaoping Lian<br>Tao Zeng, PhD

Powered by Editorial Manager® and ProduXion Manager® from Aries Systems Corporation

	Chongyang Cai
	Fushu Liu
	Zhihao Li
	Yangzi Hu
	Dongming Fang
	Weiming He
	Wangsheng Li
	Haorong Lu
	Yuxiang Li
	Huan Liu
	Xun Xu, PhD
	Ying Gu, PhD
	Fengyi Hu
	Yuliang Dong
	Sunil Kumar Sahu
**Order of Authors Secondary Information:**	
**Response to Reviewers:**	In the methods section it would be useful if you add RRID details to some of the software tools and sequencers. These needed to be listed after resources in brackets. If you are citing papers for these resources, the RRID do not replace these, and both should be included. This can be included in the methods section of the paper similar to the RRIDs included here:<br>DNBSEQ-T7 (RRID:SCR_017981); PacBio Sequel II System, RRID:SCR_017990; PLINK (RRID:SCR_001757)<br>Response: According to your suggestions, we have added RRID for the software tool and sequencer.<br>Reviewer #2: The current version of the manuscript from Guang et al on the T2T assembly of O longistaminata is a huge improvment compared to the previous version. They answered almost all questions from me and the other reviewer. For me this version is almost ok, apart for a very few things.<br>Indeed there is still an incongruency between all the sizes cited in the manuscript and revision letter: the PacBio only provides a 358Mb long genome, the mixed backbone is 343.7Mb, and the T2T was estimated to 331Mb. If authors can just explain why after cleaning for 1.27% of heterozygosity of contigs, the have such a reduction of size, it would be perfect.<br>Response: Thanks for your suggestion. If the genome is highly heterozygous, sequence reads from homologous alleles will be too different to be assembled together, and these alleles will instead be assembled separately. This means that high heterozygosity often leads assemblers to produce two copies of a region rather than one, resulting in redundancy in the assembled genome. Therefore, the PacBio HiFi genome (358 Mb) likely contains many such heterozygous regions, which contributed to its larger size. Based on the principle of Hifiasm (UL) (Chen et al., 2024, Nature Methods), the inclusion of ultra-long reads not only improves assembly contiguity but also reduces the retention of heterozygous regions in the final genome assembly. This explains why the mixed assembly is smaller (343.7 Mbp). Of the 343.7 Mbp genome, only 14 contigs could be anchored to chromosomes. The remaining unplaced short contigs (~12 Mbp) could be aligned to the mitochondrion, chloroplast, rDNA, or repeats of the Oryza genus, and were therefore filtered out. Therefore, the T2T genome size is 331Mbp. This is the same strategy that has been used on the T2T genome of maize (Chen et al., 2023, Nature Genetics), soybean ZH13-T2T (Zhang et al., 2024, The Crop Journal), T2T sandalwood (Peng et al., 2024, Gigascience), T2T Triticum aestivum L. genome (Liu et al., 2025, Nature Genetics), all with the common phenomenon that genome size was further refined from the initial assembly to the final telomere-to-telomere (T2T) version.

Powered by Editorial Manager® and ProduXion Manager® from Aries Systems Corporation

	Figure. Hybrid assembly with PacBio HiFi and ONT ultra-long reads (From Chen et al. 2024).<br><br>Reference:<br>Cheng, H., Asri, M., Lucas, J. et al. Scalable telomere-to-telomere assembly for diploid and polyploid genomes with double graph. Nat Methods 21, 967–970 (2024).<br>Chen, J., Wang, Z., Tan, K. et al. A complete telomere-to-telomere assembly of the maize genome. Nat Genet 55, 1221–1231 (2023).<br>Zhang A., Kong T., Sun B., et al. A telomere-to-telomere genome assembly of Zhonghuang 13, a widely-grown soybean variety from the original center of Glycine max. The Crop Journal 12, 142-153 (2024).<br>Liu, S., Li, K., Dai, X. et al. A telomere-to-telomere genome assembly coupled with multi-omic data provides insights into the evolution of hexaploid bread wheat. Nat Genet (2025).<br>Peng D, Hong Z, Kan S, Wu Z, Liao X. The telomere-to-telomere (T2T) genome provides insights into the evolution of specialized centromere sequences in sandalwood. Gigascience 13,2 2024.<br><br>In addition, I would ask for adding the previous comparison done with O sativa from the 1st version, in parallel with the one they performed on O. glaberrima for this version. As a matter of fact, as they stated, O. longistaminata is no more related to sativa than to glaberrima, coming from an independant line from the proto-AA genome ancestor.<br>Response: Thanks for your suggestion. We had added this in our manuscript, please see Page 6, Lines 127-138.
**Additional Information:**	
**Question**	**Response**
Are you submitting this manuscript to a special series or article collection?	No
**Experimental design and statistics**<br><br>Full details of the experimental design and statistical methods used should be given in the Methods section, as detailed in our Minimum Standards Reporting Checklist. Information essential to interpreting the data presented should be made available in the figure legends.<br><br>Have you included all the information requested in your manuscript?	Yes
**Resources**<br><br>A description of all resources used, including antibodies, cell lines, animals and software tools, with enough information to allow them to be uniquely identified, should be included in the Methods section. Authors are strongly encouraged to cite Research Resource Identifiers (RRIDs) for antibodies, model organisms and tools, where possible.	Yes

Powered by Editorial Manager® and ProduXion Manager® from Aries Systems Corporation

Have you included the information requested as detailed in our Minimum Standards Reporting Checklist?	
**Availability of data and materials**<br><br>All datasets and code on which the conclusions of the paper rely must be either included in your submission or deposited in publicly available repositories (where available and ethically appropriate), referencing such data using a unique identifier in the references and in the "Availability of Data and Materials" section of your manuscript.<br><br>Have you have met the above requirement as detailed in our Minimum Standards Reporting Checklist?	Yes

Manuscript Click here to access/download;Manuscript;Manuscript-June 4.docx

Telomere-to-telomere African wild rice (*Oryza longistaminata*) reference genome reveals segmental and structural variation

Xuanmin Guang[1,2]†, Jingnan Yang[2]†, Shilai Zhang[4]†, Fei Guo[2], Linzhou Li[1,3], Xiaoping Lian[4], Tao Zeng[2], Chongyang Cai[2], Fushu Liu[2], Zhihao Li[2], Yangzi Hu[2], Dongming Fang[1,2], Weiming He[2], Sunil Kumar Sahu[1,3], Wangsheng Li[2], Haorong Lu[2], Yuxiang Li[2], Huan Liu[1], Xun Xu[2], Ying Gu[2], Fengyi Hu[4], Yuliang Dong[2]*, Tong Wei[1,3]*

1. State Key Laboratory of Genome and Multi-omics Technologies, Key Laboratory of Genomics, Ministry of Agriculture, BGI Research, Shenzhen 518083, China

2. BGI Research, Shenzhen 518083, China.

3. BGI Research, Wuhan 430074, China

4. State Key Laboratory for Conservation and Utilization of Bio-Resources in Yunnan, Key Laboratory of Biology and Germplasm Innovation of Perennial rice (Co-construction by Ministry and Province) of Ministry of Agriculture and Rural Affairs, School of Agriculture, Yunnan University, Kunming, China

*Correspondence address: Yuliang Dong (dongyuliang@genomics.cn) and Tong Wei (weitong@genomics.cn).

†These authors should be regarded as joint first authors.

ORCID iDs: Tong Wei [0000-0002-2692-7192]; Xuanmin Guang [0000-0003-3013-634X]; Jingnan Yang [0000-0002-9738-5271]; Silai Zhang [0000-0001-9791-2468]; Sunil Kumar Sahu [0000-0002-4742-9870]; Huan Liu [0000-0003-3909-0931]; Xun Xu [0000-0002-5338-5173]; Yuliang Dong [0000-0001-8020-7420].

Abstract:

Rice (*Oryza sativa*) is one of the most important staple food crops worldwide, and its wild relatives serve as an important gene pool in its breeding. Compared with cultivated rice species, African wild rice (*Oryza longistaminata*) has several advantageous traits, such as resistance to increased biomass production, clonal propagation via rhizomes, and biotic stresses. However, previous *O. longistaminata* genome assemblies have been

hampered by gaps and incompleteness, restricting detailed investigations into their genomes. To streamline breeding endeavors and facilitate functional genomics studies, we generated a 331-Mb telomere-to-telomere (T2T) genome assembly for this species using a hybrid approach combining PacBio HiFi, Hi-C, and CycloneSEQ ultra-long reads, covering all telomeres and centromeres across the 12 chromosomes. This newly assembled genome has markedly improved over previous versions. Comparative analysis revealed a high degree of synteny with previously published genomes. A large number of structural variations were identified between *O. longistaminata*, *O. glaberrima and O.sativa.* A total of 2,466 segmentally duplicated genes were enriched in cellular amino acid metabolic processes. We detected slight expansion of some subfamilies of resistance genes and transcription factors. This newly assembled T2T genome of *O. longistaminata* provides a valuable resource for the exploration and exploitation of beneficial alleles present in wild relative species of cultivated rice.

Introduction

Rice stands as one of the world's most essential crops, serving as a staple food source for more than of the global population [1]. Rice breeding, especially wild relatives, serve as an important gene pool, is therefore critical for global food security, for which germplasms. *Oryza longistaminata* (NCBI:txid4528; 2x=2n=12), an AA genome type, thrives predominantly in the tropical regions of Western Africa, often in proximity to freshwater sources and swampy areas[2]. Although it is rarely used for human consumption, this species possesses a variety of beneficial traits. Notably, it is resistant to bacterial blight, which is linked to the *Xa21* locus[3]. Furthermore, *O. longistaminata* exhibits perennial growth and an exceptional capacity for biomass production and many efforts have been made to transfer these beneficial alleles into commercial rice varieties. In addition to contributing to breeding endeavors, *O. longistaminata* serves as a vital subject of study for investigating the genetic foundations and developmental aspects of rhizomes[4].

The assembly of a complete plant genome provides a solid basis for functional genomics investigations and facilitates the identification of candidate genes via

traditional mapping techniques. Despite the publication of several genome assembly versions, limitations stemming from sequencing technology and the intricate organization of the genome have left certain complex regions underrepresented in this reference[5, 6]. To achieve a more comprehensive representation of this fundamental reference genome, we employed a hybrid assembly strategy using Pacbio HiFi and CycloneSEQ ultra-long reads (a new single-molecule sequencer from MGI) [7] to generate backbone contigs. This marks one of the first applications of CycloneSEQ in plant T2T genomes, demonstrating its utility for complex genomic regions [7, 8]. These contigs were subsequently scaffolded into a chromosome-level assembly with the assistance of Hi-C datasets. In addition, gap filling was executed to resolve any remaining gaps. To this end, we generated a telomere-to-telomere (T2T) assembly for *O. longistaminata*, which could serve as a valuable genomic resource for future rice research and breeding.

Results and discussion

Genome assembly

We initially sequenced the genome of *O. longistaminata*, generating 27.3 Gb (~ 78 × coverage) of PacBio HiFi reads, 32 Gb (~ 100 × coverage) of Hi-C paired reads, 25.6 Gb (~71.4 × coverage) of Ultra-long CycloneSEQ reads, and 21.0 Gb (~ 60 × coverage) of MGI-Seq paired-end reads (Table S1). Using the *K-mer* method[9], we estimated the genome size of this plant to be 357 Mb, and its heterozygosity was 1.27% (Figure S1), which is similar to the size reported in previous studies[6]. Using the combined data, we first assembled a genome with a size of 343 Mb and a contig N50 of 26.02 Mb. Based on the Hi-C data, we anchored 13 contigs into 12 pseudochromosomes (Table 1, Figure S2). After that, TGS-gapcloser was employed to close the remaining gaps [10]. Finally using the seven-base telomeric repeat (CCCTAAA at the 5’ end or TTTAGGG at the 3’ end) as a sequence query, we identified all the 24 telomeres for the genome (Figure S3)[11, 12]. We then used quarTeTes to identify the centromeric regions ranging from 0.3 to 1.8 Mb on each chromosome, and assessed the regions using Hi-C data[13]. Different methods were used to evaluate the accuracy and completeness of

the assembly. First, paired-end library reads were mapped to the genome and more than 97.27% of them were aligned. Second, the BUSCO analysis indicated that the completeness of the genome reached 98.6% [14] (Table S2). Third, the LTR assembly index (LAI) value for the genome was 20.71, meeting the gold standard for genome assemblies [15]. Fourth, the calculated QV (assembly consensus quality value) using Merqury was 52.08 which indicates that the base call accuracy of the genome was greater than 99.999%[16]. As there is a previously published *O. longistaminata* genome assembly[5], we conducted a comparative gene synteny analysis at all coding sequence (CDS) levels. We subsequently identified a total of 28,627 syntenic CDS pairs through genome-wide alignment. Consistent with expectations, these two genomes displayed rather high synteny, as evidenced by the pronounced central diagonal in the alignment (Figure S4). This result demonstrated the high concordance between our assembled T2T genome and the previous *O. longistaminata* assembly.

Table 1 Summary statistics of the *O. longistaminata* genome assembly

Genome Feature	Value
Total size of assembled contigs (bp)	343,752,306
GC content	43.02%
Contig N50	26,021,309
Number of Contigs	197
Total size of assembled genomes (bp)	331,045,917
Scaffold N50	26,021,309
Complete BUSCOs	98.6%
LAI value	20.71
Repeat region	40.73%
Number of gap-free chromosomes	12
Number of candidate telomeres	24
Number of candidate centromeres	12
Number of chromosomes	12
Number of protein-coding genes	33,177
Average gene length (bp)	2439
Average exon length (bp)	261
Average intron length (bp)	384

Genome annotation

Through the utilization of *de novo* and homology-based methods, we successfully identified a total of 134 Mb of repetitive sequences in the plant genome. These repetitive sequences make up approximately 40.73% of the entire genome. Furthermore, we observed that the repeat contents were highly consistent across the entire genome as well as among the 12 pseudochromosome sequences. (Figure 1,Table S3). In this genome, LTRs and DNA transposons were the major types of repeats, accounting for approximately 20.9% and 18.5% of the whole genome, respectively. The overall repeat content was moderate, similar to the repeat content observed in other assemblies with *Oryza* genus genomes[17].

The centromere region of the genome poses a significant challenge for assembly because of its high degree of repetitive sequence content[18]. To date, the centromeric sequence of the *O. longistaminata* genome has not been fully characterized, and our new T2T genome allows deeper exploration of the repeats in these regions. The results revealed a phenomenon in which centromeric regions presented high densities of transposable elements and relatively low gene densities. Among the repeats of the centromeric regions, Gypsy elements were the most dominant type of LTR (Figure S5).

A total of 33,177 coding genes were predicted in this genome, with an average gene length of 2,439 bp and an average coding sequence (CDS) length of 1,138 bp (Figure 1, Table 1, Table S4). The functional analysis revealed that 95.74% of the coding genes could be annotated through publicly available protein datasets (Table S5), suggesting the accuracy of gene prediction.

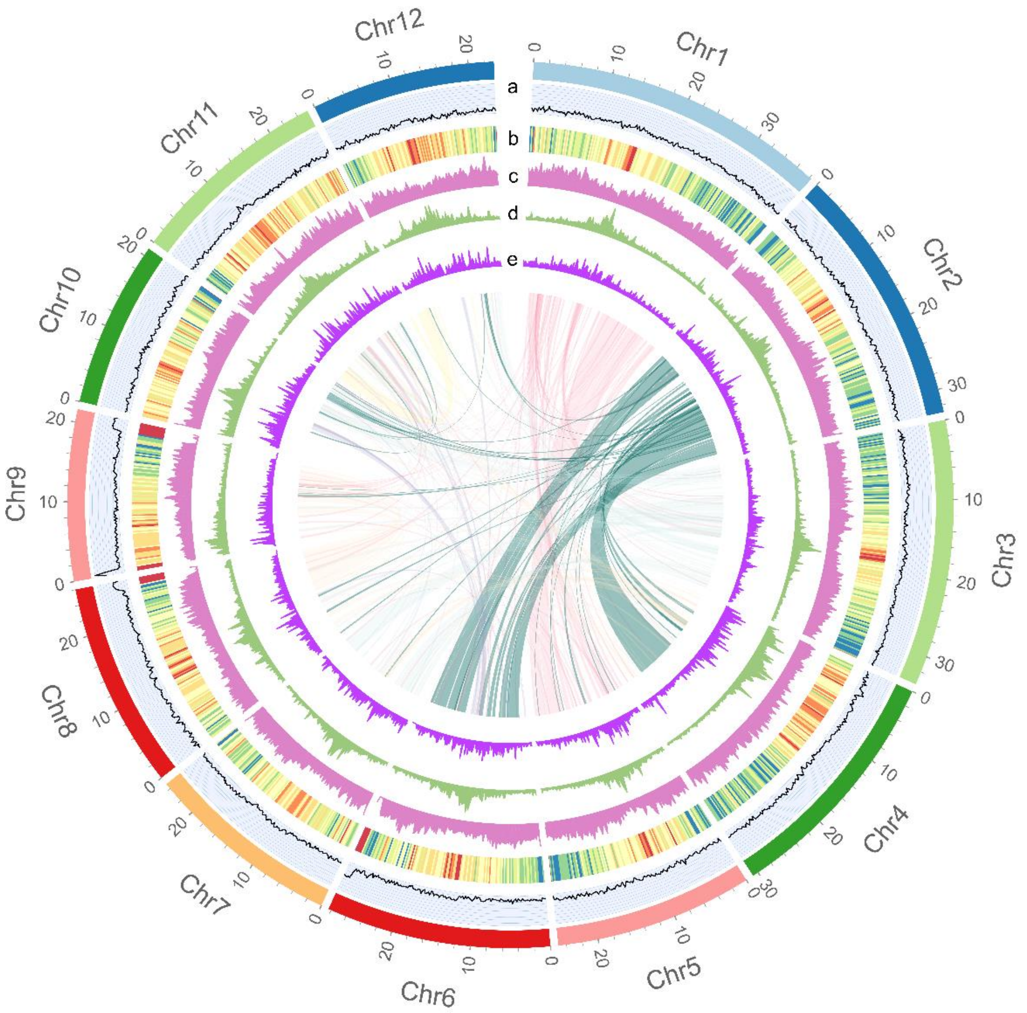

Figure 1. The telomere-to-telomere genome assembly of *O. longistaminata*. Genomic features of the *O. longistaminata* genome: (a), GC percentage (b), protein-coding genes (c), repeat sequences (d), LTR-*Gypsy* (e), and LTR-*Copia*. The collinear blocks are shown in the center.

Genome structural variations

We further performed genome-wide detection of putative structural variations (SVs) with the *O. glaberrima* (IRGC:96717) genome (Figure 2). A total of 4,790,440 single nucleotide polymorphisms (SNPs) were identified by comparing the two genomes. Among the SVs identified in our study, 198 were inversions, 8263 duplications, 8093 inverted duplications, 2667 translocations, 2663 inverted translocations (Table S6). These large SVs span more than 87 Mb throughout the entire genome, which indicates

remarkable divergence between these two species. Gene Ontology (GO) enrichment analysis of these SV-related genes revealed that they were associated with ADP binding, transposition, transposase activity and transposition, and DNA-mediated (Table S7). Furthermore, we conducted a comparative analysis with the *O. sativa* Japonica genome for SV identification (Figure 2). There were 3,738,150 SNPs, 204 inversions, 11,706 duplications, 11,175 inverted duplications, 3077 translocations, 3015 inverted translocations (Table S8). GO enrichment of these SV affected genes were related to catalytic activity, purine ribonucleotide binding, adenyl ribonucleotide binding, and telomere maintenance (Table S9).

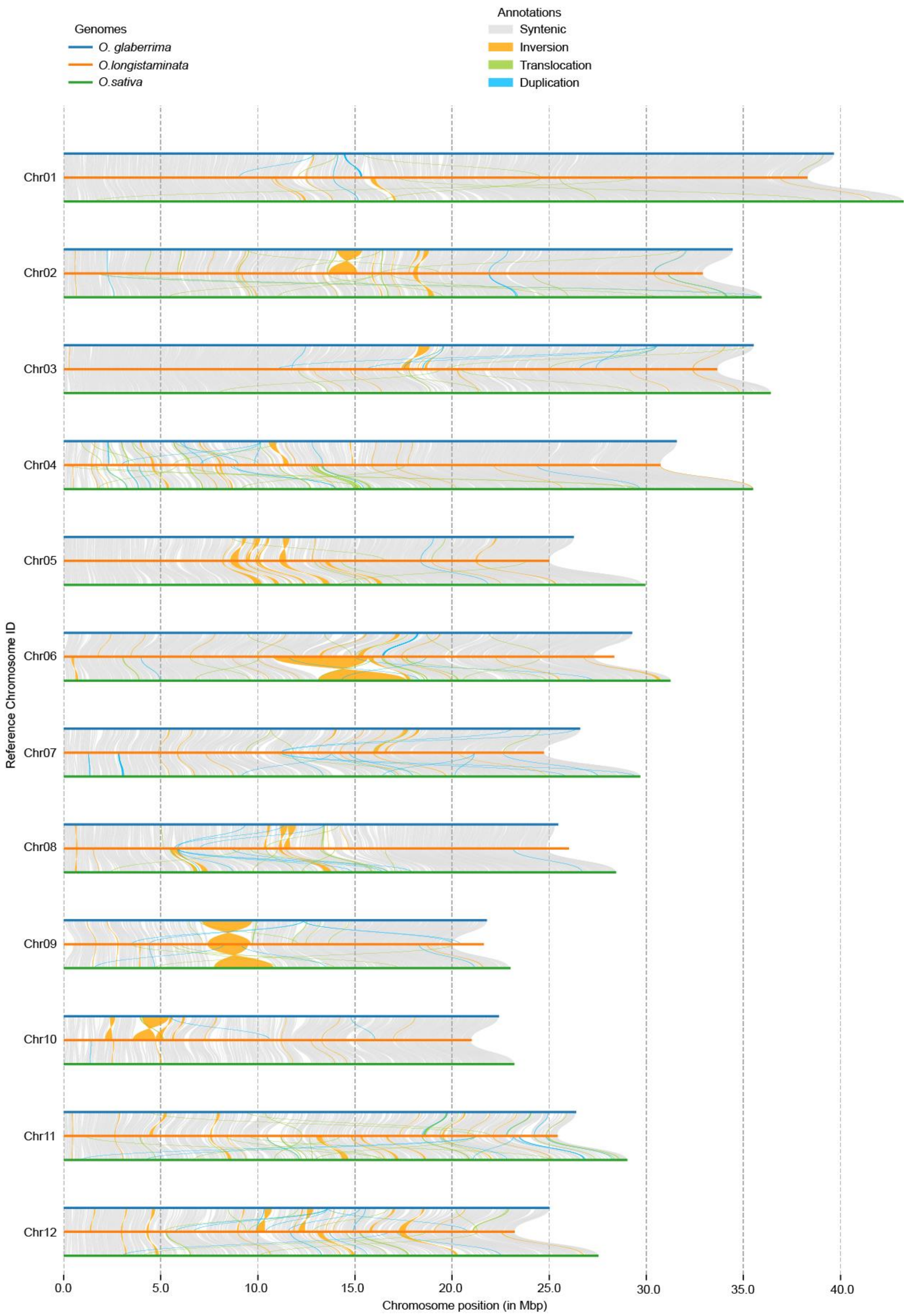

Figure 2. Collinearity and variation analysis of the T2T genome of *O. longistatminata, O.sativa* Japonica and *O. glaberrima*. The orange, blue and green lines indicate *O. longistatminata*, *O. glaberrima* and *O.sativa* Japonica, respectively.

Gray and blank blocks between various genomes indicate syntenic regions and not-aligned regions. Inversions, translocations and duplications are marked by filled orange, green and blue curves.

Analysis of SDs in the genome

Segmental Duplications (SDs) are genomic segments larger than 1 kb that repeat within the genome, and exhibit at least 90% sequence identity[19]. SDs frequently contain numerous duplicated genes, making them vital centers for gene innovation. Challenges in assembly technology have caused that the assembly of SD regions, to collapse or be entirely overlooked. As a result, this missing or inaccurate information limits our ability to understand the structure and evolution of a genome. The T2T genome of *O. longistaminata* offers an opportunity for more accurate characterization of SDs. In this study, we employed BISER to analyze the SDs in the rice genome[20], identifying 30.2 Mb of SDs, which constitute 9.12% of the genome. We discovered that SDs are not evenly distributed throughout the genome (Figure 3a). Instead, they are more frequently found on chromosomes 1 (chr1), 4 (chr4), 3 (chr3), and 2 (chr2) and less frequently found on chromosomes 9 (chr9), 10 (chr10), and 5 (chr5). Correlation analysis revealed a strong positive relationship between chromosome length and SDs ($R = 0.88$, $P = 0.00017$) (Table S10).

We proceeded to identify duplicated genes within the SD regions. Initially, we conducted an all-versus-all alignment via BLASTP[21] to identify potential paralogs, setting an E-value threshold of 10^{-5}. In the SD regions, we identified a total of 4179 pairs, of which 1233 were the top matches. For each paralogous gene pair within the SD regions, we calculated their *Ks* values as proxies for estimating the generation time of the corresponding SDs. Our findings indicate that the majority of these SDs were produced relatively recently (Ks=0.3) (Figure 3b). Gene ontology analysis revealed that these genes were significantly enriched (P<0.05) in cellular amino acid metabolic processes, carboxylic acid metabolic processes and cofactor binding (Table S11).

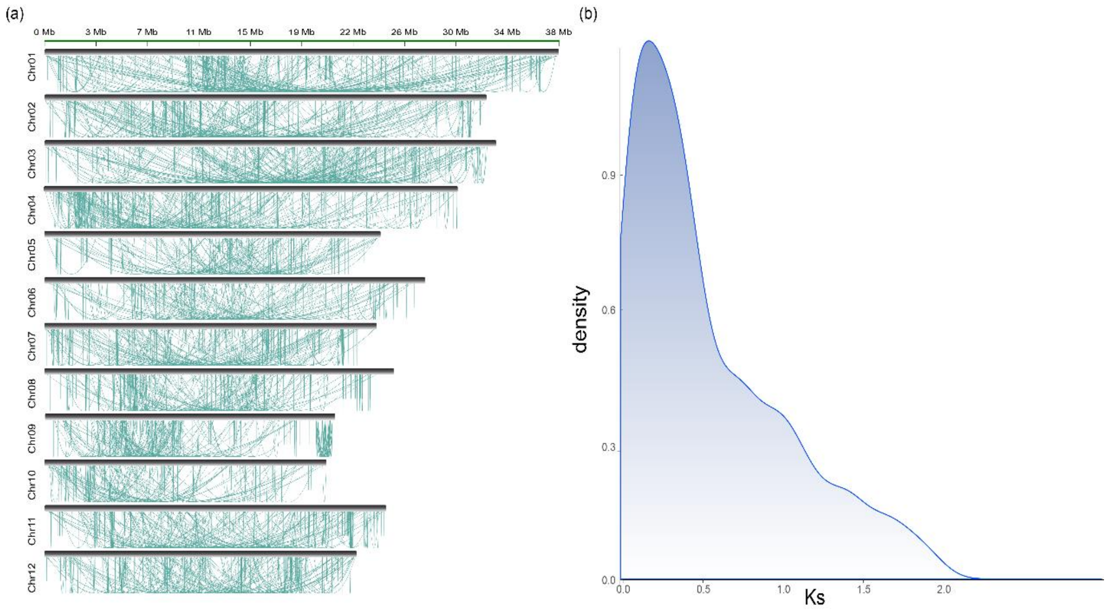

Figure 3. Segmental duplication analysis of the genome of *O. longistaminata*. (a). Distribution of intrachromosomal segmental duplication. (b) The density plot of the *Ks* value.

NBS gene family and transcription factors

Nucleotide-binding site-leucine-rich repeat (NBS-LRR) proteins, the largest family of resistance proteins, are very important for plant defense against pathogens [22, 23]. We systematically investigated the NBS-LRR genes among 11 *Oryza* species (*O.barthii*, *O. brachyantha*, *O. glaberrima*, *O. glumipatula*, *O. indica*, *O. meridionalis*, *O. nivara*, *O. punctata*, *O. rufipogon*, *O. sativa*, *O. longistaminata*) (Table S12). There were 654 NBS-LRR genes in the *O. longistaminata* genome, which were distributed in five different clusters (Table S13). Compared with other wild *Oryza* species, *O. longistaminata* has more NBS-LRR domain genes, which reflects the expansion of resistance genes in this species. NBS-LRR genes are essential components of the plant immune system, providing a mechanism for pathogen recognition and the activation of defense responses. The expansion of these types of genes in *O. longistaminata* may suggest that there was an increased ability for this species to adapt to its evolution.

We also investigated the variation in transcription factors among *Oryza* species. For *O. longistaminata,* a total of 2095 transcription factors were distributed among 86

families (Table S14). The ERF transcription factor was the most abundant (857), followed by the bHLH family (128), NAC (120), MYB (119) and C2H2 (116). Intriguingly, we found that there were 47 FAR1 genes in *O. longistaminata* which was much larger than those in other African rice accessions. Research has shown that *FAR1* performs various functions across numerous cellular processes, indicating that *FAR1* is crucial for plant growth and development[24].

Conclusion

In this study, we generated a high-quality T2T assembly of wild rice *O. longistaminata* using an integrated approach that leveraged CycloneSEQ ultra-long reads to resolve complex repetitive regions, particularly centromeres and telomeres. This hybrid strategy, combining CycloneSEQ with PacBio HiFi accuracy and Hi-C scaffolding, enabled the first complete assembly of all 24 telomeres and 12 centromeres in this species We further compared this assembly with previously published *Oryza* genomes, and identified SVs between two African wild rice accessions and *O.sativa*. Moreover, we investigated SD genes, NBS-LRR resistance genes and transcription factors. This new *O. longistaminata* assembly represents a significant update, laying fundamental evidential groundwork for focused investigations into genes associated with valuable phenotypic traits. It also sets the stage for future breeding endeavors, as well as further exploration into the evolutionary pathways of African rice and the *Oryza* genus.

Methods

Material preparation and sequencing

Fresh young leaves from mature *O. longistaminata* plants were collected from Yunnan University, Yunnan Province, China. Genomic DNA was extracted for Pacbio HiFi, MGI CycloneSeq and MGIseq (DNBSEQ-T7) (RRID:SCR_017981). Pair-end libraries 500 bp in length were constructed and sequenced on the MGIseq platform. For Cyclone sequencing, genomic DNA was extracted via the CTAB method[25], and the CycloneSEQ library was generated following the manufacturer’s guidelines. Each

sample, comprising 2 μg of input DNA (≥21 ng/μL), was initially diluted with nuclease-free water to a total volume of 192 μL, followed by mixing with 14 μL of DNA repair buffer 1, 14 μL of DNA repair buffer 2, 12 μL of DNA repair enzyme 1, and 8 μL of DNA repair enzyme 2. The mixtures were then incubated in a thermocycler through the following steps: 10 min at 20°C, 10 min at 65°C, and held at 4°C. After incubation, the mixes were purified using a 1.0x volume of DNA clean beads, and DNAs were eluted with 240 μL of nuclease-free water. Next, the purified end-repaired samples were mixed with 10 μL sequencing adaptors, 100 μL 4x ligation buffer, 40 μL DNA ligase, and 10 μL of nuclease-free water before being incubated at 25°C for 30 minutes to complete the adaptor ligation. The ligated products were again purified with a volume of 1.0x DNA clean beads and long fragment wash buffer was applied to gently resuspend the beads. After removing the supernatant, the libraries were recovered in 42 μL of elution buffer and quantified on a Qubit fluorometer[26]. Each prepared library was sequenced on the CycloneSEQ (WuTong02) platform according to the protocol[27]. A total of 25.6 Gb of clean subreads longer than 79 kb were obtained and used as Ultra-long reads. For the construction of the PacBio HiFi library, more than 5 μg of DNA was prepared for size selection using a BluePippin (RRID:SCR_020505) instrument. Subsequently, PacBio Sequel II single-molecule real-time (SMRT) bell libraries of approximately 20 kb were constructed in accordance with the PacBio protocol. The library was loaded into SMRT cells with a DNA Sequencing Reagent Kit. These SMRT cells were then run on a PacBio Sequel II CCS system (RRID:SCR_017990), which generated 24 Gb of long-read sequencing data.

Genome assembly

We employed hifiasm (RRID:SCR_021069, v 0.19.5-r592) for genome assembly, utilizing both HiFireads and ultra-long CycloneSeq reads under the mixed assembly model with default settings[28]. Subsequent polishing of the assembled genome was performed NextPolish (RRID:SCR_025232) [29] with MGISEQ reads (RRID:SCR_017981). For chromosomal anchoring of the contigs, we first utilized cleaned HiC reads. Unique mapping reads were identified using bowtie2

(RRID:SCR_016368, v 2.3.2) [30], followed by the detection of valid interacting paired reads via Juicer (RRID:SCR_017226, v 2.8.1) [31]. These valid read pairs were then used to construct pseudo-chromosome sequences with 3D-DNA[32]. The HiC interactions are shown as heatmaps through Juicebox (RRID:SCR_021172) [33]. After that, the genome had only one gap. Finally, gap filling was accomplished by using TGS-Gapcloser (RRID:SCR_017633, v1.2.1)[10] with CycloneSeq reads, and corrections were made by using pilon (RRID:SCR_014731, v 1.24) with MGI paired-end reads[34]. Based on the genome of *O. sativa*, we artificially reoriented some chromosomes and renamed their chromosome numbers.

To access the quality of the assemblies, we mapped the short paired-end reads to the assembly by using the BWA-MEM tool from BWA (RRID:SCR_022192) [35], and BUSCO analysis was performed with the embryophyte_odb9 database (RRID:SCR_015008) [14].

Genome annotation

We initially generated repetitive libraries for both species through a dual approach involving homology comparison and de novo prediction. Repetitive sequences were identified using LTR Finder (RRID:SCR_015247) [36] and RepeatModeler (RRID:SCR_015027) [37]. Homology-based prediction was performed using TRF (RRID:SCR_022193) [38] and RepeatMasker (RRID:SCR_012954) [39] with the Repbase TE library. The annotated and classified repetitive sequences were then used to mask the genomes with RepeatMasker. Additionally, we employed LTR Retriever[40], in conjunction with LTR Finder, to calculate the LTR Assembly Index (LAI), which assesses assembly continuity by evaluating the assembly of repeat sequences[15].

For the RNA-Seq assisted predictions, ISO-seq sequences were obtained from a mixed tissue. We employed SMRT Link v8.0, applying the parameters –min-passes 3, –min-length 50, –max-length 15000, and –min-rq 0.99, to refine the circular consensus sequence (CCS) subreads, subsequently gathering high-quality reads. For classifying the full-length reads, Lima (RRID:SCR_025520, v2.2.0) was utilized with the

following settings: –isoseq, –dump-clips, and –peak-guess[41]. The final assembly of full-length Iso-seq transcripts was achieved using isoseq3 (RRID:SCR_022749)[42], which employs the refine module (parameters: –require-polya and –min-polya-length 20) and the cluster module (parameters: –verbose and –use-qvs). After that, Transdecode (RRID:SCR_017647) [43] was used to predict the CDS and the longest CDSs were fed into Maker for gene annotation. For homologous predictions, protein sequences from *Zea mays*, *O. sativa*, and *A.thaliana* were used. The MAKER2 (RRID:SCR_005309) pipeline [44] was used for protein-coding gene annotation, and de novo gene models were accessed via AUGUSTUS (RRID:SCR_008417) [45] and Fgenesh (RRID:SCR_011928) [46]. To predict gene functions, we performed a BLAST search of their protein sequences against the Swiss-Prot and NR databases, using a threshold E-value of 1e-5. We subsequently employed InterProScan (RRID:SCR_005829) to annotate motifs and domains by searching for matches in those databases[47].

Structural variation analysis

We utilized a suite of tools from MUMmer4 (RRID:SCR_018171) [48] to analyze genomic differences between *O. longistaminata* and *O. glaberrima*. The nucmer tool was employed to compare syntenic chromosomes, and the results were subsequently filtered through a delta-filter with the parameters '-c 100 -b 500 -l 50'. The alignments were then converted into tab-delimited files using the show-coords program. Finally, SyRI (RRID:SCR_023008) was applied to identify structural variations (SVs) [49].

Identification of telomeres and centromeres

In most plants, telomere sequences consist of short, conserved satellite repeats arranged in tandem. We identified a typical plant telomere sequence (CCCTAAA at the 5' end or TTTAGGG at the 3' end) and subsequently identified telomeres across all 12 chromosomes by using tidk [12]. To detect centromeric regions, we employed the quartet (RRID:SCR_025258) tool[13]. This tool is well suited for identifying genomic areas with high and low gene densities, as well as short tandem repeats, which are characteristic features of centromeric regions.

Detection of Segmental Duplications (SDs)

Briefly, our genome assembly underwent an initial soft-mask process, during which all common and tandem repeats were converted into lowercase letters. Following this, BISER was employed to detect Segmental Duplications (SDs), utilizing its default parameters[20].

Ks **of the duplicated gene pairs**

Protein sequences from duplicated gene pairs within SDs were extracted and subsequently aligned utilizing the MUSCLE (RRID:SCR_011812) alignment program[50]. These aligned protein sequences were then transformed into corresponding coding sequence alignments through PAL2NAL[51]. Next, we calculated the rate of synonymous substitutions per synonymous site (*Ks*) for each gene pair, employing the KaKs_Calculator (RRID:SCR_022068) [52]. The distribution of *Ks* values was graphically plotted and visualized using the R statistical software.

Identification of *NBS-LRR* genes and TF genes

For each species analyzed, protein sequences were extracted and subsequently screened against the raw Hidden Markov Model (HMM) of the NB-ARC family (PF00931) utilizing HMMER (RRID:SCR_005305, v 3.1b1), applying the default parameters[53]. NBS-specific HMMs were constructed utilizing the hmmbuild program within HMMER, which were then employed to identify NBS-encoding proteins. To identify specific protein domains, PfamScan (RRID:SCR_004726) was utilized for screening these proteins against the Pfam-A database (Pfam31.0) [54]. Additionally, coiled-coil domains were detected using the ncoils tool [55], with its default parameters applied. The iTAK (Integrated Toolkit for Analysis of Kinase) software tool was used to identify transcription factors (TFs) and protein kinases (PKs) among the Oryza genus species[56].

Acknowledgements

This work was supported by the State Key Laboratory of Genome and Multi-omics Technologies, Guangdong Provincial Key Laboratory of core collection of crop genetic

resources research and application, Shenzhen Engineering laboratory of Crop Molecular design breeding, the National Natural Science Foundation of China (32322063 to Shilai Zhang) and the Shenzhen Science and Technology Program (KQTD20221101093603011 to Jingnan Yang). This work is a part of the 10KP project, and is also supported by the China National GeneBank.

Conflicts of interest statement

WT., Y.D., J.Y. H.Liu, F.G., T.Z., H.Lu, C.C., F.L.,Z.L.,Y.H., W.L., L.Y.,X.X., W.H., Y.G., S.K.S, X.G., L.L. and D.F. are employees of the BGI Group (as that has helped R&D of the MGI sequencers).

Authors' contributions

WT. and Y.D. conceived the project and were responsible for the project initiation. J.Y. and Y.D. designed the experiments. S.Z., H.Liu, X.L., and F.H. contributed to sample preparation. F.G., T.Z., H.Lu, C.C., F.L.,Z.L.,Y.H., W.L., L.Y.,X.X., and Y.G performed the experiments and sequencing. X.G., L.L., D.F., J.Y. and W.H. performed the data analysis. X.G. wrote the original draft. T.W., S.K.S., and Y.D. revised the manuscript. All authors read and approved the manuscript.

Data availability

The T2T genome assembly and CycloneSeq reads have been deposited to NCBI and CNSA (CNGB Nucleotide Sequence Archive) with BioProject accessions PRJNA1259753 and CNP0005176, respectively. All additional supporting data are available in the *GigaScience* repository, GigaDB [57].

References

1. Fukagawa NK, Ziska LH: **Rice: Importance for Global Nutrition.** *J Nutr Sci Vitaminol (Tokyo)* 2019, **65:**S2-s3.
2. Vaughan DA: *The wild relatives of rice: a genetic resources handbook.* Int. Rice Res. Inst.; 1994.
3. Song WY, Wang GL, Chen LL, Kim HS, Pi LY, Holsten T, Gardner J, Wang B, Zhai WX, Zhu LH, et al: **A receptor kinase-like protein encoded by the rice disease resistance gene, Xa21.** *Science* 1995, **270:**1804-1806.
4. Li W, Zhang S, Huang G, Huang L, Zhang J, Li Z, Hu F: **A Genetic Network Underlying Rhizome Development in Oryza longistaminata.** *Front Plant*

Sci 2022, **13:**866165.
5. Reuscher S, Furuta T, Bessho-Uehara K, Cosi M, Jena KK, Toyoda A, Fujiyama A, Kurata N, Ashikari M: **Assembling the genome of the African wild rice Oryza longistaminata by exploiting synteny in closely related Oryza species.** *Communications Biology* 2018, **1:**162.
6. Zhang Y, Zhang S, Liu H, Fu B, Li L, Xie M, Song Y, Li X, Cai J, Wan W, et al: **Genome and Comparative Transcriptomics of African Wild Rice Oryza longistaminata Provide Insights into Molecular Mechanism of Rhizomatousness and Self-Incompatibility.** *Molecular Plant* 2015, **8:**1683-1686.
7. Zhang J-Y, Zhang Y, Wang L, Guo F, Yun Q, Zeng T, Yan X, Yu L, Cheng L, Wu W, et al: **A single-molecule nanopore sequencing platform.** *bioRxiv* 2024**:**2024.2008.2019.608720.
8. Liang H, Zou Y, Wang M, Hu T, Wang H, He W, Ju Y, Guo R, Chen J, Guo F, et al: **Efficiently constructing complete genomes with CycloneSEQ to fill gaps in bacterial draft assemblies.** *GigaByte* 2025, **2025:**gigabyte154. https://doi.org/10.1101/2024.09.05.611410.
9. Vurture GW, Sedlazeck FJ, Nattestad M, Underwood CJ, Fang H, Gurtowski J, Schatz MC: **GenomeScope: fast reference-free genome profiling from short reads.** *Bioinformatics* 2017, **33:**2202-2204.
10. Xu M, Guo L, Gu S, Wang O, Zhang R, Peters BA, Fan G, Liu X, Xu X, Deng L, Zhang Y: **TGS-GapCloser: A fast and accurate gap closer for large genomes with low coverage of error-prone long reads.** *GigaScience* 2020, **9:**giaa094.
11. Song J-M, Xie W-Z, Wang S, Guo Y-X, Koo D-H, Kudrna D, Gong C, Huang Y, Feng J-W, Zhang W, et al: **Two gap-free reference genomes and a global view of the centromere architecture in rice.** *Molecular Plant* 2021, **14:**1757-1767.
12. Brown MR, Manuel Gonzalez de La Rosa P, Blaxter M: **tidk: a toolkit to rapidly identify telomeric repeats from genomic datasets.** *Bioinformatics* 2025, **41**.
13. Lin Y, Ye C, Li X, Chen Q, Wu Y, Zhang F, Pan R, Zhang S, Chen S, Wang X, et al: **quarTeT: a telomere-to-telomere toolkit for gap-free genome assembly and centromeric repeat identification.** *Horticulture Research* 2023, **10:**uhad127.
14. Simão FA, Waterhouse RM, Ioannidis P, Kriventseva EV, Zdobnov EM: **BUSCO: assessing genome assembly and annotation completeness with single-copy orthologs.** *Bioinformatics* 2015, **31:**3210-3212.
15. Ou S, Chen J, Jiang N: **Assessing genome assembly quality using the LTR Assembly Index (LAI).** *Nucleic Acids Res* 2018, **46:**e126.
16. Rhie A, Walenz BP, Koren S, Phillippy AM: **Merqury: reference-free quality, completeness, and phasing assessment for genome assemblies.** *Genome Biology* 2020, **21:**245.
17. Qin P, Lu H, Du H, Wang H, Chen W, Chen Z, He Q, Ou S, Zhang H, Li X, et

al: **Pan-genome analysis of 33 genetically diverse rice accessions reveals hidden genomic variations.** *Cell* 2021, **184:**3542-3558.e3516.

18. Deng Y, Liu S, Zhang Y, Tan J, Li X, Chu X, Xu B, Tian Y, Sun Y, Li B, et al: **A telomere-to-telomere gap-free reference genome of watermelon and its mutation library provide important resources for gene discovery and breeding.** *Mol Plant* 2022, **15:**1268-1284.
19. Bailey JA, Yavor AM, Massa HF, Trask BJ, Eichler EE: **Segmental duplications: organization and impact within the current human genome project assembly.** *Genome Res* 2001, **11:**1005-1017.
20. Išerić H, Alkan C, Hach F, Numanagić I: **Fast characterization of segmental duplication structure in multiple genome assemblies.** *Algorithms for Molecular Biology* 2022, **17:**4.
21. Altschul SF, Gish W, Miller W, Myers EW, Lipman DJ: **Basic local alignment search tool.** *J Mol Biol* 1990, **215:**403-410.
22. DeYoung BJ, Innes RW: **Plant NBS-LRR proteins in pathogen sensing and host defense.** *Nat Immunol* 2006, **7:**1243-1249.
23. Shao ZQ, Xue JY, Wang Q, Wang B, Chen JQ: **Revisiting the Origin of Plant NBS-LRR Genes.** *Trends Plant Sci* 2019, **24:**9-12.
24. Ma L, Li G: **FAR1-RELATED SEQUENCE (FRS) and FRS-RELATED FACTOR (FRF) Family Proteins in Arabidopsis Growth and Development.** *Front Plant Sci* 2018, **9:**692.
25. Sahu SK, Thangaraj M, Kathiresan K: **DNA Extraction Protocol for Plants with High Levels of Secondary Metabolites and Polysaccharides without Using Liquid Nitrogen and Phenol.** *ISRN Mol Biol* 2012, **2012:**205049.
26. Wang M, Liang H, Zeng T, Chen J, Zou Y, Xiao L: **CycloneSEQ library construction from DNA of isolated bacteria.** protocols.io 2025. https://dx.doi.org/10.17504/protocols.io.rm7vzk3k2vx1/v1
27. Liang H, Wang M, Chen J, Zhang Y, Zeng T, Zou Y, Xiao L: **CycloneSEQ sequencing protocol for bacterial libraries.** protocols.io 2025. https://dx.doi.org/10.17504/protocols.io.rm7vz6n6rgx1/v1
28. Cheng H, Concepcion GT, Feng X, Zhang H, Li H: **Haplotype-resolved de novo assembly using phased assembly graphs with hifiasm.** *Nature Methods* 2021, **18:**170-175.
29. Hu J, Fan J, Sun Z, Liu S: **NextPolish: a fast and efficient genome polishing tool for long-read assembly.** *Bioinformatics* 2020, **36:**2253-2255.
30. Langmead B, Salzberg SL: **Fast gapped-read alignment with Bowtie 2.** *Nat Methods* 2012, **9:**357-359.
31. Durand NC, Shamim MS, Machol I, Rao SS, Huntley MH, Lander ES, Aiden EL: **Juicer Provides a One-Click System for Analyzing Loop-Resolution Hi-C Experiments.** *Cell Syst* 2016, **3:**95-98.
32. Dudchenko O, Batra SS, Omer AD, Nyquist SK, Hoeger M, Durand NC, Shamim MS, Machol I, Lander ES, Aiden AP, Aiden EL: **De novo assembly of the Aedes aegypti genome using Hi-C yields chromosome-length scaffolds.** *Science* 2017, **356:**92-95.

33. Durand NC, Robinson JT, Shamim MS, Machol I, Mesirov JP, Lander ES, Aiden EL: **Juicebox Provides a Visualization System for Hi-C Contact Maps with Unlimited Zoom.** *Cell Syst* 2016, **3:**99-101.
34. Walker BJ, Abeel T, Shea T, Priest M, Abouelliel A, Sakthikumar S, Cuomo CA, Zeng Q, Wortman J, Young SK, Earl AM: **Pilon: an integrated tool for comprehensive microbial variant detection and genome assembly improvement.** *PLoS One* 2014, **9:**e112963.
35. Li H, Durbin R: **Fast and accurate short read alignment with Burrows–Wheeler transform.** *Bioinformatics* 2009, **25:**1754-1760.
36. Xu Z, Wang H: **LTR_FINDER: an efficient tool for the prediction of full-length LTR retrotransposons.** *Nucleic Acids Res* 2007, **35:**W265-268.
37. Flynn JM, Hubley R, Goubert C, Rosen J, Clark AG, Feschotte C, Smit AF: **RepeatModeler2 for automated genomic discovery of transposable element families.** *Proceedings of the National Academy of Sciences* 2020, **117:**9451-9457.
38. Benson G: **Tandem repeats finder: a program to analyze DNA sequences.** *Nucleic Acids Res* 1999, **27:**573-580.
39. **RepeatMasker Open-4.0** [<http://www.repeatmasker.org>]
40. Ou S, Jiang N: **LTR_retriever: A Highly Accurate and Sensitive Program for Identification of Long Terminal Repeat Retrotransposons.** *Plant Physiol* 2018, **176:**1410-1422.
41. Ritchie ME, Phipson B, Wu D, Hu Y, Law CW, Shi W, Smyth GK: **limma powers differential expression analyses for RNA-sequencing and microarray studies.** *Nucleic Acids Res* 2015, **43:**e47.
42. Gordon SP, Tseng E, Salamov A, Zhang J, Meng X, Zhao Z, Kang D, Underwood J, Grigoriev IV, Figueroa M, et al: **Widespread Polycistronic Transcripts in Fungi Revealed by Single-Molecule mRNA Sequencing.** *PLoS One* 2015, **10:**e0132628.
43. **TransDecoder/TransDecoder, GitHub. (n.d.).** [https://github.com/TransDecoder/TransDecoder]
44. Holt C, Yandell M: **MAKER2: an annotation pipeline and genome-database management tool for second-generation genome projects.** *BMC Bioinformatics* 2011, **12:**491.
45. Stanke M, Morgenstern B: **AUGUSTUS: a web server for gene prediction in eukaryotes that allows user-defined constraints.** *Nucleic Acids Res* 2005, **33:**W465-467.
46. Salamov AA, Solovyev VV: **Ab initio gene finding in Drosophila genomic DNA.** *Genome Res* 2000, **10:**516-522.
47. Jones P, Binns D, Chang HY, Fraser M, Li W, McAnulla C, McWilliam H, Maslen J, Mitchell A, Nuka G, et al: **InterProScan 5: genome-scale protein function classification.** *Bioinformatics* 2014, **30:**1236-1240.
48. Marçais G, Delcher AL, Phillippy AM, Coston R, Salzberg SL, Zimin A: **MUMmer4: A fast and versatile genome alignment system.** *PLoS Comput Biol* 2018, **14:**e1005944.

49. Goel M, Sun H, Jiao WB, Schneeberger K: **SyRI: finding genomic rearrangements and local sequence differences from whole-genome assemblies.** *Genome Biol* 2019, **20:**277.
50. Edgar RC: **MUSCLE: multiple sequence alignment with high accuracy and high throughput.** *Nucleic Acids Res* 2004, **32:**1792-1797.
51. Suyama M, Torrents D, Bork P: **PAL2NAL: robust conversion of protein sequence alignments into the corresponding codon alignments.** *Nucleic Acids Res* 2006, **34:**W609-612.
52. Wang D, Zhang Y, Zhang Z, Zhu J, Yu J: **KaKs_Calculator 2.0: a toolkit incorporating gamma-series methods and sliding window strategies.** *Genomics Proteomics Bioinformatics* 2010, **8:**77-80.
53. Finn RD, Clements J, Eddy SR: **HMMER web server: interactive sequence similarity searching.** *Nucleic Acids Res* 2011, **39:**W29-37.
54. Punta M, Coggill PC, Eberhardt RY, Mistry J, Tate J, Boursnell C, Pang N, Forslund K, Ceric G, Clements J, et al: **The Pfam protein families database.** *Nucleic Acids Res* 2012, **40:**D290-301.
55. Lupas A, Van Dyke M, Stock J: **Predicting coiled coils from protein sequences.** *Science* 1991, **252:**1162-1164.
56. Zheng Y, Jiao C, Sun H, Rosli HG, Pombo MA, Zhang P, Banf M, Dai X, Martin GB, Giovannoni JJ, et al: **iTAK: A Program for Genome-wide Prediction and Classification of Plant Transcription Factors, Transcriptional Regulators, and Protein Kinases.** *Mol Plant* 2016, **9:**1667-1670.
57. Guang X, Yang J, Zhang S, Guo F, Li L, Lian X, Zeng T, Cai C, Liu F, Li Z, et al: **Supporting data for "Telomere-to-telomere African wild rice (Oryza longistaminata) reference genome reveals segmental and structural variation".** GigaScience Database; 2025. https://doi.org/10.5524/102693.

Supplementary Figures

Click here to access/download

Supplementary Material

Supplementary Figures.docx

Supplementary Tables

Click here to access/download
Supplementary Material
Supplementary Table.xlsx

Personal Cover

Click here to access/download;Personal Cover;Cover letter 407.docx

Dear Editor,

Thank you very much for reviewing our Manuscript GIGA-D-24-00479R1 entitled: "**Telomere-to-telomere African wild rice (*Oryza longistaminata*) reference genome reveals segmental and structural variation**", which we hope will be considered for publication as a **Data note** in ***Gigascience***.

The comments of the reviewers were highly insightful and enabled us to greatly improve the quality of our manuscript. We revised our manuscript as reviewers' comments. In this version, we added the previous comparison done with *O. sativa* from the 1st version, according to the reviewer's suggestion. According to the editor's suggestion, we also added RRID details to some of the software tools and sequencers. We highlighted texts that involve changes in the main article in yellow color.

We prepared the point-by-point responses to each of the comments and suggestions of reviewers. We hope that these revisions in the manuscript and our accompanying responses are sufficient to make our manuscript suitable for publication in ***Gigascience***.

We look forward to hearing from you at your earliest convenience.

Sincerely,

Tong Wei, PhD.

Email: weitong@genomics.cn

BGI Research, Wuhan, China.

Point to Point to Reviewer

Reviewer #2: The current version of the manuscript from Guang et al on the T2T assembly of O longistaminata is a huge improvment compared to the previous version. They answered almost all questions from me and the other reviewer. For me this version is almost ok, apart for a very few things.

Indeed there is still an incongruency between all the sizes cited in the manuscript and revision letter: the PacBio only provides a 358Mb long genome, the mixed backbone is 343.7Mb, and the T2T was estimated to 331Mb. If authors can just explain why after cleaning for 1.27% of heterozygosity of contigs, the have such a reduction of size, it would be perfect. Response: Thanks for your suggestion. If the genome is highly heterozygous, sequence reads from homologous alleles will be too different to be assembled together, and these alleles will instead be assembled separately. This means that high heterozygosity often leads assemblers to produce two copies of a region rather than one, resulting in redundancy in the assembled genome. Therefore, the PacBio HiFi genome (358 Mb) likely contains many such heterozygous regions, which contributed to its larger size. Based on the principle of Hifiasm (UL) (Chen et al., 2024, *Nature Methods*), the inclusion of ultra-long reads not only improves assembly contiguity but also reduces the retention of heterozygous regions in the final genome assembly. This explains why the mixed assembly is smaller (343.7 Mbp). Of the 343.7 Mbp genome, only 14 contigs could be anchored to chromosomes. The remaining unplaced short contigs (~12 Mbp) could be aligned to the mitochondrion, chloroplast, rDNA, or repeats of the *Oryza* genus, and were therefore filtered out. Therefore, the T2T genome size is 331Mbp. This is the same strategy that has been used on the T2T genome of maize (Chen et al., 2023, *Nature Genetics*), soybean ZH13-T2T (Zhang et al., 2024, *The Crop Journal*), T2T sandalwood (Peng et al., 2024, *Gigascience*), T2T Triticum aestivum L. genome (Liu et al., 2025, *Nature Genetics*), all with the common phenomenon that genome size was further refined from the initial assembly to the final telomere-to-telomere (T2T) version.

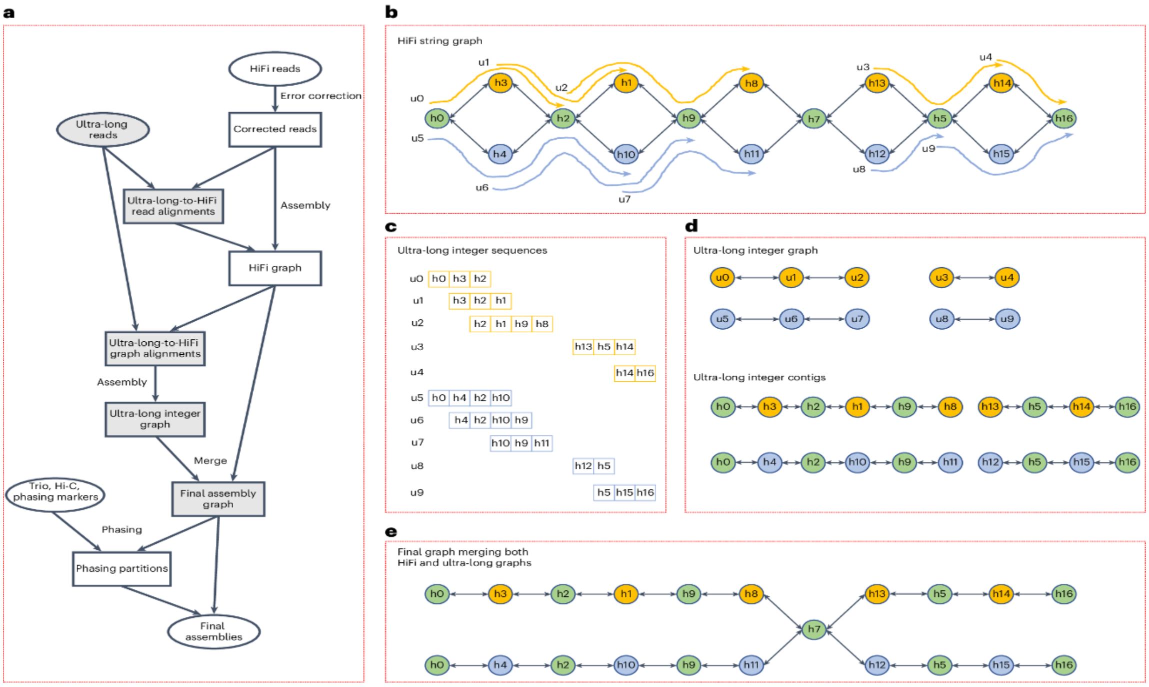

Figure. Hybrid assembly with PacBio HiFi and ONT ultra-long reads (From Chen et al. 2024).

Reference:

Cheng, H., Asri, M., Lucas, J. et al. Scalable telomere-to-telomere assembly for diploid and polyploid genomes with double graph. *Nat Methods* 21, 967–970 (2024).
Chen, J., Wang, Z., Tan, K. et al. A complete telomere-to-telomere assembly of the maize genome. Nat Genet 55, 1221–1231 (2023).
Zhang A., Kong T., Sun B., et al. A telomere-to-telomere genome assembly of Zhonghuang 13, a widely-grown soybean variety from the original center of Glycine max. The Crop Journal 12, 142-153 (2024).
Liu, S., Li, K., Dai, X. et al. A telomere-to-telomere genome assembly coupled with multi-omic data provides insights into the evolution of hexaploid bread wheat. Nat Genet (2025).
Peng D, Hong Z, Kan S, Wu Z, Liao X. The telomere-to-telomere (T2T) genome provides insights into the evolution of specialized centromere sequences in sandalwood. Gigascience 13,2 2024.

In addition, I would ask for adding the previous comparison done with O sativa from the 1st version, in parallel with the one they performed on O. glaberrima for this version. As a matter of fact, as they stated, O. longistaminata is no more related to sativa than to glaberrima, coming from an independant line from the proto-AA genome ancestor.

Response: Thanks for your suggestion. We had added this in our manuscript, please see Page 6, Lines 127-138.
