# Supplementary material for: Telomere-to-telomere African wild rice (Oryza longistaminata) reference genome reveals segmental and structural variation
Source: Gigascience. 2025 Aug 19;14:giaf074. doi: 10.1093/gigascience/giaf074 (PMC12360840; doi:10.1093/gigascience/giaf074)
Supplement: giaf074_Authors_Response_To_Reviewer_Comments_Original_Submission [file giaf074_authors_response_to_reviewer_comments_original_submission.pdf]

Author's Response To Reviewer Comments

Response to Reviewer comments
Reviewer #1: The authors generated a 343-Mb telomere-to-telomere (T2T) genome assembly for an African wild rice (Oryza longistaminata), covering all telomeres and centromeres across the 12 chromosomes, and performed genome annotation and analyses on structural variations and NLR genes. While the manuscript has provided a valuable genome sequence, several problems should be addressed before the manuscript can be published.
Response: Thank you for reviewing our manuscript, and providing constructive suggestions.
Major issues
1. The authors estimated that the genome heterozygosity is 1.27%, which is quite high, so I am wondering how large the assembled genome size is using only HiFi data, which could reflect the actual heterozygosity rate of the genome, particularly by comparing it with the final genome size of 12 chromosomes. If there was only one gap in the initial assembly of Hifiasm (a total of 13 contigs), it is unlikely that the genome has such a high heterozygosity. In Table 1, the total size of assembled genome was 331,045,917bp. If this is the summed size of 12 chromosomes, it should be used as the final genome size in the main text. Please clarify. Also, what is the base accuracy of Ultra-long CycloneSEQ data? which is useful to readers for this is a new sequencing technology.
Response: According to your suggestion, we used only HiFi data to assemble the genome, resulting in a genome size of 358 Mbp. This is slightly larger than our mixed-assembled contig backbone (343.7 Mbp). In the initial assembly generated by Hifiasm, there were 197 contigs. Based on the Hi-C data, we observed that most of the long contigs (>10 Mbp) were clustered into separate chromosomes. Additionally, the last two contigs presented Hi-C interaction signals, supporting the conclusion that they should be connected and form part of the same chromosome. The heterozygosity rate of 1.27% was estimated based on k-mer analysis of the WGS sequencing data, which is independent of the assembly process. This rate reflects the overall genetic diversity within the genome, but it does not necessarily translate directly into assembly fragmentation. Instead, it may manifest as duplicated regions or alternate haplotigs, which were resolved during the final assembly steps, and it may also benefit the acquisition of the two haplotypes of this genome. The total size of the T2T genome was determined to be 331 Mbp, and we have updated this information in the main text. According to a previous report (Zhang et al., BioRxiv, 2024), the overall per-base error rate for CycloneSEQ data was estimated to be 3.94%. Given its sequencing mechanism, we believe that the same base error rate applies to ultra-long reads.
Reference:
Zhang J-Y, Zhang Y, Wang L, Guo F, Yun Q, Zeng T, Yan X, Yu L, Cheng L, Wu W, et al: A single-molecule nanopore sequencing platform. bioRxiv 2024:2024.2008.2019.608720.

2. For SV detection, considering that the assembled genome in the manuscript (does it have a accession ID or name?) is an African wild rice, it is rather strange that the authors did not compare it with an O. glaberrima genome, but with an O. sativa genome. Meanwhile, the name of the genomes should be mentioned since there were so many different genomes in each species, all with different SV variations between them.
Response: We thank the reviewer for pointing out the need for clarification regarding the O. longistaminata genome assembly. The assembled genome of O. longistaminata (also known as red rice) is indeed the same accession that was published by Zhang et al. (Molecular Plant, 2015), its accession number is unknown and was kindly supplied by Prof. Dr. Hiroshi Hyakutake which was derived from the Ministry of Agriculture and Forestry, Japan. In our study, we selected O. sativa japonica as the primary reference for comparison because it is the most widely used reference genome and serves as a well-established model for rice genomics. Based on the phylogenetic relationship reported by Zhang et al. (Molecular Plant, 2015), O. sativa and O. glaberrima form a sister group, making O. sativa japonica a relevant comparator for initial analyses. Following the reviewer's suggestion, we also performed a comparative analysis with O. glaberrima (
IRGC:96717) to identify structural variations (SVs). This additional comparison provided further insights into the genomic differences between these species, as detailed on Page 6, Lines 125–138 of the revised manuscript. We have clearly stated the names and sources of all the genomes used in our study. We appreciate the reviewer's comment and have ensured that the manuscript now includes all necessary

details regarding the genome assemblies and comparisons.
Reference:
Zhang Y, Zhang S, Liu H, Fu B, Li L, Xie M, Song Y, Li X, Cai J, Wan W, Kui L, Huang H, Lyu J, Dong Y, Wang W, Huang L, Zhang J, Yang Q, Shan Q, Li Q, Huang W, Tao D, Wang M, Chen M, Yu Y, Wing RA, Wang W, Hu F. Genome and Comparative Transcriptomics of African Wild Rice Oryza longistaminata Provide Insights into Molecular Mechanism of Rhizomatousness and Self-Incompatibility. Mol Plant. 2015,8(11):1683-6.

3. The conclusion that "This distribution suggests that chromosomes 1, 4, 3, and 2 might have contributed to the evolution of rice in previously unrecognized ways (Table S8)" is purely speculative, and thus should be removed from the manuscript, or the authors should provide more evidence to support it.
Response: Yes, we agree with you and this sentence has been removed.
4. The author claimed that "Compared with other Oryza species, O. longistaminata has many fewer NBS-lRR domain genes, which reflects a contraction of resistance genes in this species." Please give specific gene numbers for each species. Meanwhile, the conclusion does not look right here since it looks that O. longistaminata had more NBS-LRR genes than other species.
Response: We thank the reviewer for his valuable suggestion. The number of NBS genes identified in our study can be found in Table S10. Our analysis revealed that O. longistaminata possesses a greater number of NBS-LRR genes compared to other wild rice species do. We have revised the relevant sentence in the manuscript to reflect this finding. Please refer to Page 9, Lines 178–180 for the updated text.

Minor issues
1. What is "quartets"?
Response: This was a spelling mistake; it should be quarTeT which is a software used for T2T genome assembly. We have revised this term.
2. The author used "11 Oryza species" which included O. indica, please clarify what this species is.
Response: O.indica is Oryza sativa Indica Group (long-grained rice) 9311.

Reviewer #2: The manuscript from Guang et al deals with a T2T assembly for the wild perennial African rice Oryza longistaminata. Using last up to date technologies and approaches, authors provided a high quality assembly for this wild species, rending it a valuable ressource for understanding rice evolution. While the results as assembly are of high quality, the interpretation of some biological results, in particular about the NBS-LRR, are quite weird, in my opinion, and need to be more refined. That's why I think the manuscript should be published, but after major corrections.
Response: Thank you for reviewing our manuscript, and providing useful suggestions
in details:

-Introduction: not sure the exceptional biomass is a good idea from longistaminata, as this plant has avery high content in silicium, rendering its biomass complex to use.
Response: Yes, we agree with your point. However, exceptional biomass production is a distinctive characteristic of O. longistaminata. Although its biomass is difficult to utilize directly, it may hold potential for molecular breeding applications.
- Methods: We do not have access to most of the command options and command-lines. please provide them at least as a texte file in supp data. In addition, some of the references for tools are missing. Finally, please provide the accession number of the assembled plant.
Response: We thank the editor for their request prior to review. As instructed, we uploaded all major result files and command scripts to the FTP website. The files are organized and accessible for review. Please check the FTP site at (files.gigadb.org) for the complete set of data and scripts. If any additional information or clarification is needed, we are happy to provide further details. We checked throughout the entire manuscript and added the missing reference. The assembled genome of O. longistaminata (also known as red rice) is indeed the same accession that was published by Zhang et al. (Molecular Plant, 2015); its accession number is unknown and was kindly supplied by Prof. Dr. Hiroshi Hyakutake which was derived from the Ministry of Agriculture and Forestry, Japan.
Reference:
Zhang Y, Zhang S, Liu H, Fu B, Li L, Xie M, Song Y, Li X, Cai J, Wan W, Kui L, Huang H, Lyu J, Dong Y, Wang W, Huang L, Zhang J, Yang Q, Shan Q, Li Q, Huang W, Tao D, Wang M, Chen M, Yu Y, Wing RA, Wang W, Hu F. Genome and Comparative Transcriptomics of African Wild Rice Oryza longistaminata

Provide Insights into Molecular Mechanism of Rhizomatousness and Self-Incompatibility. Mol Plant. 2015,8(11):1683-6.

- Assembly in itself: O longistaminata is a outcrossing heterozygous organism. Did you obtained the two haplotypes ?
Response: We appreciate the reviewer's insightful question regarding the heterozygous nature of O. longistaminata. As an outcrossing species, O. longistaminata indeed exhibits high heterozygosity. In our study, we aimed to carry out analysis of the T2T genome of this strain. We used PacBio HiFi long reads with HIC data and Ultra long Nanopore reads, which are highly accurate and suitable for resolving haplotypes in heterozygous genomes. The reads were assembled using Hifiasm, which is designed to separate haplotypes during the assembly process. The assembly resulted in two primary contigs, representing the major haplotype, and one alternate contig, representing the secondary haplotype. In the assembly process, the assembled graph of primary contigs, which includes a complete assembly with long stretches of phased blocks, was used as the backbone for the T2T genome.

- Comparison with the previous longistaminata genome: is the inversion in middle of Chr6 specific ? or due to an error of previous assembly ?
Response: We reviewed the assembly process and reported that Chr6 consists of only one initial contig. We mapped HiFi reads to this chromosome and examined its coverage, confirming that there were no abnormal regions. Additionally, we compared the whole genome of O. glaberrima and observed no inversion of Chr6. Therefore, we infer that this is an error of the previous assembly.
- Table 1: what do you mean "Total size of assembled genomes (bp) 331,045,917" ? What is the residual percentage of N ?
Response: This is the genome size of this gap-free T2T assembly. So, there isn’t any N region for this genome.
- Figure 1 and others: please show the legend in other way, here we may mix it with the main text. in addition, check the legends for spelling and the size of figure (3b eg) for lisibility
Response: Thank your for suggestion, we have revised this.
- Syri/MUMmer analysis: you limit as min size at 1kb ? What was the order of query vs ref ? can we have a bed file with the positions ?
Response: Yes, we set the minimum size threshold at 1 kb. Initially, we used O. longistaminata as the query and O. sativa japonica as the reference. Following Reviewer #1's suggestion, we compared our genome to that of O. glaberrima, which is now the reference. The VCF results from this analysis are available for download from the FTP site.
- SD: is there a statistical link between chromosome size and number of SD ? It could explain why the first 4 ones have more SD. In general, the data are missing stats.
Response: Thank you for your suggestion. We performed a correlation analysis between chromosome size and the number of SDs, which revealed a strong positive correlation ($R = 0.88$, $P = 0.00017$). This result suggests that larger chromosomes tend to have more SDs, which may explain why the first four chromosomes contain a greater number of SDs. We have revised the manuscript accordingly. Please see page 8 lines 155-157.
- GO in SD: any statistical validation ?
Response: The enrichment of GO terms with P values lesser than 0.05. Please see page 8 line 165 and Table S9.
- Genomes comparison: please provide the acc number of the genome you used for comparison.
Response: We have provided the genome accession number and please see it in the table below.
Table R1. Accession numbers of the Oryza genus genome
O.barthii O.brachyantha O.glaberrima O.glumipatula O.indica O.meridionalis O.nivara O.punctata O.rufipogon O.sativa
Accession number GCA_000182155.2 GCA_000231095.2 GCA_000147395.1 GCA_000576495.1 GCA_000004655.2 GCA_000338895.2 GCA_000576065.1 GCA_000573905.1 GCA_000817225.1 GCA_001433935.1

- NBS-LRR: the longistaminata genome has 215 genes for 116 to 289 for other oryza so I cannot see any contraction or expansion. in addition, the text here is weird, starting speaking of onctraction then going to expansion ???
Response: We appreciate the reviewer's careful reading. We apologize that there was an error in our

original description of these sentences. We have revised this to “Compared with other wild Oryza species, O. longistaminata has more NBS-LRR domain genes, which reflects a expansion of resistance genes in this species.” Please see page 9 lines 178-180.
- TF analysis; the african assemblies are quite bad I think, explaining the discrepency. For glaberrima, did you check the one from Tranchant-Dubreuil et al, 2023 ?
Response: We noticed that the genome assembly of African rice was fragmented. Therefore, we used a new genome assembly version downloaded from NCBI (https://www.ncbi.nlm.nih.gov/datasets/genome/GCF_000147395.1/), which was generated from PacBio long reads and assembled with Canu software. The O. glaberrima genome has a size of 347.3 Mbp with a contig N50 of 15.2 Mbp, indicating high genome quality and continuity.

Editorial Manager®

Cookie Preference Center

We use cookies which are necessary to make our site work. We may also use additional cookies to analyze, improve and personalize our content and your digital experience. For more information, see our Cookie Policy.

You may choose not to allow some types of cookies. However, blocking some types may impact your experience of our site and the services we are able to offer. See the different category headings below to find out more or change your settings.

Allow all

Manage Consent Preferences

Strictly Necessary Cookies

Always active

These cookies are necessary for the website to function and cannot be switched off in our systems. They are usually only set in response to actions made by you which amount to a request for services, such as setting your privacy preferences, logging in or filling in forms. You can set your browser to block or alert you about these cookies, but some parts of the site will not then work.

Functional Cookies

These cookies enable the website to provide enhanced functionality and personalisation. They may be set by us or by third party providers whose services we have added to our pages. If you do not allow these cookies then some or all of these services may not function properly.

Performance Cookies

These cookies allow us to count visits and traffic sources so we can measure and improve the performance of our site. They help us to know which pages are the most and least popular and see how visitors move around the site.

Targeting Cookies

These cookies may be set through our site by our advertising partners. They may be used by those companies to build a profile of your interests and show you relevant adverts on other sites. If you do not allow these cookies, you will experience less targeted advertising.

Cookie List

Clear

Apply Cancel

Consent Leg.Interest

Confirm my choices

Powered by onetrus
