# Supplementary material for: Telomere-to-telomere African wild rice (Oryza longistaminata) reference genome reveals segmental and structural variation
Source: Gigascience. 2025 Aug 19;14:giaf074. doi: 10.1093/gigascience/giaf074 (PMC12360840; doi:10.1093/gigascience/giaf074)
Supplement: giaf074_Authors_Response_To_Reviewer_Comments_Revision_1 [file giaf074_authors_response_to_reviewer_comments_revision_1.pdf]

Author's Response To Reviewer Comments

In the methods section it would be useful if you add RRID details to some of the software tools and sequencers. These needed to be listed after resources in brackets. If you are citing papers for these resources, the RRID do not replace these, and both should be included. This can be included in the methods section of the paper similar to the RRIDs included here:
DNBSEQ-T7 (RRID:SCR_017981); PacBio Sequel II System, RRID:SCR_017990; PLINK (RRID:SCR_001757)
Response: According to your suggestions, we have added RRID for the software tool and sequencer.
Reviewer #2: The current version of the manuscript from Guang et al on the T2T assembly of O longistaminata is a huge improvment compared to the previous version. They answered almost all questions from me and the other reviewer. For me this version is almost ok, apart for a very few things. Indeed there is still an incongruency between all the sizes cited in the manuscript and revision letter: the PacBio only provides a 358Mb long genome, the mixed backbone is 343.7Mb, and the T2T was estimated to 331Mb. If authors can just explain why after cleaning for 1.27% of heterozygosity of contigs, the have such a reduction of size, it would be perfect.
Response: Thanks for your suggestion. If the genome is highly heterozygous, sequence reads from homologous alleles will be too different to be assembled together, and these alleles will instead be assembled separately. This means that high heterozygosity often leads assemblers to produce two copies of a region rather than one, resulting in redundancy in the assembled genome. Therefore, the PacBio HiFi genome (358 Mb) likely contains many such heterozygous regions, which contributed to its larger size. Based on the principle of Hifiasm (UL) (Chen et al., 2024, Nature Methods), the inclusion of ultra-long reads not only improves assembly contiguity but also reduces the retention of heterozygous regions in the final genome assembly. This explains why the mixed assembly is smaller (343.7 Mbp). Of the 343.7 Mbp genome, only 14 contigs could be anchored to chromosomes. The remaining unplaced short contigs (~12 Mbp) could be aligned to the mitochondrion, chloroplast, rDNA, or repeats of the Oryza genus, and were therefore filtered out. Therefore, the T2T genome size is 331Mbp. This is the same strategy that has been used on the T2T genome of maize (Chen et al., 2023, Nature Genetics), soybean ZH13-T2T (Zhang et al., 2024, The Crop Journal), T2T sandalwood (Peng et al., 2024, Gigascience), T2T Triticum aestivum L. genome (Liu et al., 2025, Nature Genetics), all with the common phenomenon that genome size was further refined from the initial assembly to the final telomere-to-telomere (T2T) version.

Figure. Hybrid assembly with PacBio HiFi and ONT ultra-long reads (From Chen et al. 2024).

Reference:
Cheng, H., Asri, M., Lucas, J. et al. Scalable telomere-to-telomere assembly for diploid and polyploid genomes with double graph. Nat Methods 21, 967–970 (2024).
Chen, J., Wang, Z., Tan, K. et al. A complete telomere-to-telomere assembly of the maize genome. Nat Genet 55, 1221–1231 (2023).
Zhang A., Kong T., Sun B., et al. A telomere-to-telomere genome assembly of Zhonghuang 13, a widely-grown soybean variety from the original center of Glycine max. The Crop Journal 12, 142-153 (2024).
Liu, S., Li, K., Dai, X. et al. A telomere-to-telomere genome assembly coupled with multi-omic data provides insights into the evolution of hexaploid bread wheat. Nat Genet (2025).
Peng D, Hong Z, Kan S, Wu Z, Liao X. The telomere-to-telomere (T2T) genome provides insights into the evolution of specialized centromere sequences in sandalwood. Gigascience 13,2 2024.

In addition, I would ask for adding the previous comparison done with O sativa from the 1st version, in parallel with the one they performed on O. glaberrima for this version. As a matter of fact, as they stated, O. longistaminata is no more related to sativa than to glaberrima, coming from an independant line from the proto-AA genome ancestor.
Response: Thanks for your suggestion. We had added this in our manuscript, please see Page 6, Lines 127-138.

Cookie Preference Center

We use cookies which are necessary to make our site work. We may also use additional cookies to analyze, improve and personalize our content and your digital experience. For more information, see our Cookie Policy.

You may choose not to allow some types of cookies. However, blocking some types may impact your experience of our site and the services we are able to offer. See the different category headings below to find out more or change your settings.

Allow all

Manage Consent Preferences

Strictly Necessary Cookies

Always active

These cookies are necessary for the website to function and cannot be switched off in our systems. They are usually only set in response to actions made by you which amount to a request for services, such as setting your privacy preferences, logging in or filling in forms. You can set your browser to block or alert you about these cookies, but some parts of the site will not then work.

Functional Cookies

These cookies enable the website to provide enhanced functionality and personalisation. They may be set by us or by third party providers whose services we have added to our pages. If you do not allow these cookies then some or all of these services may not function properly.

Performance Cookies

These cookies allow us to count visits and traffic sources so we can measure and improve the performance of our site. They help us to know which pages are the most and least popular and see how visitors move around the site.

Targeting Cookies

These cookies may be set through our site by our advertising partners. They may be used by those companies to build a profile of your interests and show you relevant adverts on other sites. If you do not allow these cookies, you will experience less targeted advertising.

Cookie List

Clear

Apply Cancel
Consent Leg.Interest

Confirm my choices

Powered by onetrust
