# Supplementary material for: Telomere-to-telomere African wild rice (Oryza longistaminata) reference genome reveals segmental and structural variation
Source: Gigascience. 2025 Aug 19;14:giaf074. doi: 10.1093/gigascience/giaf074 (PMC12360840; doi:10.1093/gigascience/giaf074)
Supplement: giaf074_Reviewer_1_Report_Original_Submission — Chengzhi Liang -- 11/29/2024 [file giaf074_reviewer_1_report_original_submission.pdf]

Reviewer Report

Title: Telomere-to-telomere African wild rice (Oryza longistaminata) reference genome reveals segmental and structural variation

Version: Original Submission **Date:** 11/29/2024

Reviewer name: Chengzhi Liang

Reviewer Comments to Author:

The authors generated a 343-Mb telomere-to-telomere (T2T) genome assembly for an African wild rice (Oryza longistaminata), covering all telomeres and centromeres across the 12 chromosomes, and performed genome annotation and analyses on structural variations and NLR genes. While the manuscript has provided a valuable genome sequence, several problems should be addressed before the manuscript can be published.Major issues1. The authors estimated that the genome heterozygosity is 1.27%, which is quite high, so I am wondering how large the assembled genome size is using only HiFi data, which could reflect the actual heterozygosity rate of the genome, particularly by comparing it with the final genome size of 12 chromosomes. If there was only one gap in the initial assembly of Hifiasm (a total of 13 contigs), it is unlikely that the genome has such a high heterozygosity. In Table 1, the total size of assembled genome was 331,045,917bp. If this is the summed size of 12 chromosomes, it should be used as the final genome size in the main text. Please clarify. Also, what is the base accuracy of Ultra-long CycloneSEQ data? which is useful to readers for this is a new sequencing technology.2. For SV detection, considering that the assembled genome in the manuscript (does it have a accession ID or name?) is an African wild rice, it is rather strange that the authors did not compare it with an O. glaberrima genome, but with an O. sativa genome. Meanwhile, the name of the genomes should be mentioned since there were so many different genomes in each species, all with different SV variations between them.3. The conclusion that "This distribution suggests that chromosomes 1, 4, 3, and 2 might have contributed to the evolution of rice in previously unrecognized ways (Table S8)" is purely speculative, and thus should be removed from the manuscript, or the authors should provide more evidence to support it.4. The author claimed that "Compared with other Oryza species, O. longistaminata has many fewer NBS-lRR domain genes, which reflects a contraction of resistance genes in this species." Please give specific gene numbers for each species. Meanwhile, the conclusion does not look right here since it looks that O. longistaminata had more NBS-LRR genes than other species.Minor issues1. What is "quartets"?2. The author used "11 Oryza species" which included O. indica, please clarify what this species is.

Level of Interest

Please indicate how interesting you found the manuscript: Choose an item.

Quality of Written English

Please indicate the quality of language in the manuscript: Choose an item.

Declaration of Competing Interests

Please complete a declaration of competing interests, considering the following questions:

- Have you in the past five years received reimbursements, fees, funding, or salary from an organisation that may in any way gain or lose financially from the publication of this manuscript, either now or in the future?
- Do you hold any stocks or shares in an organisation that may in any way gain or lose financially from the publication of this manuscript, either now or in the future?
- Do you hold or are you currently applying for any patents relating to the content of the manuscript?
- Have you received reimbursements, fees, funding, or salary from an organization that holds or has applied for patents relating to the content of the manuscript?
- Do you have any other financial competing interests?
- Do you have any non-financial competing interests in relation to this paper?

If you can answer no to all of the above, write 'I declare that I have no competing interests' below. If your reply is yes to any, please give details below.

I declare that I have no competing interests

I agree to the open peer review policy of the journal. I understand that my name will be included on my report to the authors and, if the manuscript is accepted for publication, my named report including any attachments I upload will be posted on the website along with the authors' responses. I agree for my report to be made available under an Open Access Creative Commons CC-BY license (http://creativecommons.org/licenses/by/4.0/). I understand that any comments which I do not wish to be included in my named report can be included as confidential comments to the editors, which will not be published.

Choose an item.

To further support our reviewers, we have joined with Publons, where you can gain additional credit to further highlight your hard work (see: https://publons.com/journal/530/gigascience). On publication of this paper, your review will be automatically added to Publons, you can then choose whether or not to claim your Publons credit. I understand this statement.

Yes Choose an item.
