# Supplementary material for: Telomere-to-telomere African wild rice (Oryza longistaminata) reference genome reveals segmental and structural variation
Source: Gigascience. 2025 Aug 19;14:giaf074. doi: 10.1093/gigascience/giaf074 (PMC12360840; doi:10.1093/gigascience/giaf074)
Supplement: giaf074_Reviewer_2_Report_Original_Submission — Francois Sabot -- 12/19/2024 [file giaf074_reviewer_2_report_original_submission.pdf]

Reviewer Report

Title: Telomere-to-telomere African wild rice (Oryza longistaminata) reference genome reveals segmental and structural variation

Version: Original Submission **Date:** 12/19/2024

Reviewer name: Francois Sabot

Reviewer Comments to Author:

The manuscript from Guang et al deals with a T2T assembly for the wild perennial African rice Oryza longistaminata. Using last up to date technologies and approaches, authors provided a high quality assembly for this wild species, rending it a valuable ressource for understanding rice evolution.While the results as assembly are of high quality, the interpretation of some biological results, in particular about the NBS-LRR, are quite weird, in my opinion, and need to be more refined. That's why I think the manuscript should be published, but after major corrections.in details:-Introduction: not sure the exceptional biomass is a good idea from longistaminata, as this plant has avery high content in silicium, rendering its biomass complex to use.- Methods: We do not have access to most of the command options and command-lines. please provide them at least as a texte file in supp data. In addition, some of the references for tools are missing. Finally, please provide the accession number of the assembled plant.- Assembly in itself: O longistaminata is a outcrossing heterozygous organism. Did you obtained the two haplotypes ?- Comparison with the previous longistaminata genome: is the inversion in middle of Chr6 specific ? or due to an error of previous assembly ?- Table 1: what do you mean "Total size of assembled genomes (bp) 331,045,917" ? What is the residual percentage of N ?- Figure 1 and others: please show the legend in other way, here we may mix it with the main text. in addition, check the legends for spelling and the size of figure (3b eg) for lisibility- Syri/MUMmer analysis: you limit as min size at 1kb ? What was the order of query vs ref ? can we have a bed file with the positions ?- SD: is there a statistical link between chromosome size and number of SD ? It could explain why the first 4 ones have more SD. In general, the data are missing stats.- GO in SD: any statistical validation ?- Genomes comparison: please provide the acc number of the genome you used for comparison.- NBS-LRR: the longistaminata genome has 215 genes for 116 to 289 for other oryza so I cannot see any contraction or expansion. in addition, the text here is weird, starting speaking of onctraction then going to expansion ???- TF analysis; the african assemblies are quite bad I think, explaining the discrepency. For glaberrima, did you check the one from Tranchant-Dubreuil et al, 2023 ?

Level of Interest

Please indicate how interesting you found the manuscript: Choose an item.

Quality of Written English

Please indicate the quality of language in the manuscript: Choose an item.

Declaration of Competing Interests

Please complete a declaration of competing interests, considering the following questions:

- Have you in the past five years received reimbursements, fees, funding, or salary from an organisation that may in any way gain or lose financially from the publication of this manuscript, either now or in the future?
- Do you hold any stocks or shares in an organisation that may in any way gain or lose financially from the publication of this manuscript, either now or in the future?
- Do you hold or are you currently applying for any patents relating to the content of the manuscript?
- Have you received reimbursements, fees, funding, or salary from an organization that holds or has applied for patents relating to the content of the manuscript?
- Do you have any other financial competing interests?
- Do you have any non-financial competing interests in relation to this paper?

If you can answer no to all of the above, write 'I declare that I have no competing interests' below. If your reply is yes to any, please give details below.

I declare that I have no competing interests

I agree to the open peer review policy of the journal. I understand that my name will be included on my report to the authors and, if the manuscript is accepted for publication, my named report including any attachments I upload will be posted on the website along with the authors' responses. I agree for my report to be made available under an Open Access Creative Commons CC-BY license (http://creativecommons.org/licenses/by/4.0/). I understand that any comments which I do not wish to be included in my named report can be included as confidential comments to the editors, which will not be published.

Choose an item.

To further support our reviewers, we have joined with Publons, where you can gain additional credit to further highlight your hard work (see: https://publons.com/journal/530/gigascience). On publication of this paper, your review will be automatically added to Publons, you can then choose whether or not to claim your Publons credit. I understand this statement.

Yes Choose an item.
